# K739 is preferentially targeted over K725 in the deSUMOylation process of neuronal nitric oxide synthase

**DOI:** 10.3389/fchem.2025.1672437

**Published:** 2025-09-04

**Authors:** Yuxin Xu, Wei Wan, Nan Wang

**Affiliations:** 1 Jiangsu Key Laboratory of Brain Disease Bioinformation, Research Center for Biochemistry and Molecular Biology, Xuzhou Medical University, Xuzhou, Jiangsu, China; 2 National Demonstration Center for Experimental Basic Medical Science Education, Xuzhou Medical University, Xuzhou, Jiangsu, China

**Keywords:** nNOS, peptides (pep), DeSUMOylation, SENP1, molecular dynamics simulation

## Abstract

**Introduction:**

Neuronal nitric oxide synthase (nNOS) produces nitric oxide (NO) in neurons, essential for learning and memory, but excessive activity causes oxidative/nitrosative stress, contributing to neuropsychiatric disorders. nNOS activation is regulated by calcium-activated calmodulin (CaM) binding and SUMO1 modification at the CaM-binding domain (CaMBD). Our prior studies showed modified CaMBD peptides can modulate NO production in mouse neurons, but their *in vivo* efficacy, particularly in the middle cerebral artery occlusion (MCAO) model, remains untested. The overlap between SUMO1 and CaM-binding sites raises questions about their interplay and the role of SENP1-mediated deSUMOylation in attenuating nNOS hyperactivity. This study investigates the interactions between CaMBD peptides, SUMO1 modification at K725 and K739, and SENP1-mediated deSUMOylation to develop therapeutic strategies for regulating nNOS activity and mitigating neurotoxicity.

**Methods:**

Structural models of the SENP1-SUMO1-nNOS complex were built using X-ray crystallographic data (PDB: 2IY0, 2LL7) and homology modeling, followed by molecular docking with Z-DOCK and 500-ns molecular dynamics simulations using AMBER 24 with the Amber19SB force field. Binding free energies were calculated via MM-GBSA, and interactions analyzed with CPPTRAJ. *In vivo*, male C57BL/6 mice (4–6 weeks) underwent MCAO. Peptides (25 μg/mouse) were injected into hippocampal CA1 and cortical M1 regions pre-MCAO. Spatial learning and memory were assessed via the Morris water maze, and infarct volumes quantified by TTC staining 24 h post-MCAO. Data were analyzed using one-way ANOVA.

**Results:**

Peptides N0 and N3 showed no significant toxicity, while N1 and N2 reduced survival, likely due to excessive nNOS activation and inflammation. N0 reduced infarct volume but did not improve behavioral outcomes. Molecular dynamics simulations revealed distinct deSUMOylation mechanisms at K725 and K739, with K739 showing stronger SENP1 binding, supported by RMSD and RMSF analyses. Free energy calculations confirmed SENP1’s binding selectivity at K739.

**Discussion:**

N0 mitigated ischemia-induced damage in the MCAO model, unlike N2 and N3, suggesting moderate CaMBD affinity prevents excessive nNOS activation and aberrant SUMOylation at K739, critical for neuroprotection. Stronger SENP1 binding at K739 supports targeted deSUMOylation strategies. Further research is needed to optimize peptide therapies and clarify CaM-SUMOylation interactions for nNOS-related disorders.

## Introduction

Neuronal nitric oxide synthase (nNOS), one of three nitric oxide synthase (NOS) isoforms alongside inducible (iNOS) and endothelial (eNOS) subtypes, catalyzes the conversion of L-arginine to nitric oxide (NO) in the presence of NADPH and tetrahydrobiopterin (BH4) cofactors (Palmer et al., 1988). Exclusively active in its dimeric form, nNOS is constitutively expressed in mammalian brain and skeletal muscle, serving as the primary neuronal NO source critical for learning, memory, and muscle contraction (Baldelli et al., 2014; Ito et al., 2013; James et al., 2015; Kelley et al., 2009; Kelley et al., 2011; Pavesi et al., 2013). However, overactivation of nNOS in neurons can result in oxidative stress and build-up of free radicals, causing neurotoxicity. Neurotoxicity is linked to neuronal death in neurological and psychiatric illnesses, such as ischemic stroke, traumatic brain injury, and hypoxic-ischemic encephalopathy. Therefore, clarification of the structural underpinnings of the nNOS activation pathway is crucial to understand its physiological and pathological significance (Qu et al., 2020; Yin et al., 2013; Yu et al., 2008; Zhou et al., 2010). The nNOS monomer comprises a PDZ domain, an oxygenase domain, a calmodulin-binding domain (CaMBD), and a reductase domain, with the CaMBD (residues 725–745) playing a pivotal role in regulating nNOS activity through conformational and covalent modifications (Zhou and Zhu, 2009).

Regulation of the catalytic activity of nNOS can be divided into two types, namely conformational regulation and covalent modification, both of which can occur in the CaMBD region. Calmodulin (CaM) is a conformational activator of nNOS that effectively enhances its catalytic activity. Its mechanism and mode of action have been extensively studied till date. To investigate nNOS regulation, we previously designed interfering peptides based on the rat nNOS-CaMBD sequence (residues 731–744, designated N0). Three modified peptides—N1 (L734F mutation), N2 (F731Y, F740Y mutations), and N3 (F731L, V738L, F740L mutations)—were developed to modulate CaM binding and explore the impact of amino acid polarity and hydrophobicity on nNOS activation (Wan and Wang, 2024). These peptides target the CaMBD to competitively inhibit calmodulin (CaM) binding, which enhances nNOS catalytic activity via calcium-mediated interactions (Abu-Soud et al., 1994; Bredt and Snyder, 1990). While our prior studies demonstrated that N2 and N3 increase NO production in cultured neurons, their *in vivo* effects remain untested. The middle cerebral artery occlusion (MCAO) model in mice is essential to evaluate these peptides’ neuroprotective potential, as it replicates ischemic stroke conditions, enabling assessment of their ability to mitigate brain tissue damage and behavioral deficits caused by nNOS overactivation (Cheng and Wang, 2022).

SUMOylation, a covalent post-translational modification involving small ubiquitin-like modifier (SUMO) proteins, is an emerging mechanism for sustaining nNOS hyperactivity (Du et al., 2020; Wang and Hou, 2020). SUMO1, activated by E1 and E2 enzymes and catalyzed by the E3 ligase PIAS3, covalently binds to lysine residues K725 and K739 within the nNOS-CaMBD, promoting prolonged NO release and neurotoxicity. Unlike CaM-mediated activation, SUMOylation is less reversible, contributing to persistent nNOS activity. Our preliminary findings suggest that K739 exhibits stronger SUMO1 binding than K725, potentially due to differences in local amino acid environments, though the full PIAS3 interaction domain remains unclear. The overlap between SUMO1 and CaM binding sites raises critical questions about competitive interactions, necessitating studies on how N0/N1/N2/N3 peptides influence SUMO1 modification and vice versa. The deSUMOylation enzyme SENP1 counteracts SUMOylation by cleaving SUMO1 from nNOS, restoring basal activity (Daniel et al., 2017). Using the SENP1-SUMO1 crystal structure (PDB: 2IY0), we constructed models linking K725 or K739 to SUMO1’s C-terminal glycine, with SENP1 positioned to assess deSUMOylation selectivity. Molecular dynamics simulations revealed preferential SENP1 binding to K739, suggesting a prioritized deSUMOylation mechanism.

This study integrates *in vivo* experiments using the MCAO model in mice with molecular dynamics simulations to evaluate the neuroprotective effects of CaMBD-derived peptides (N0, N1, N2, N3) and elucidate the selectivity of SENP1-mediated deSUMOylation at K725 and K739 sites. Anticipated results include differential peptide toxicity and diffusion in brain tissue, partial morphological neuroprotection by N0 without behavioral improvements, preferential SENP1 binding to the K739 site driven by enhanced electrostatic interactions, hydrogen bonding, and binding free energies, and limited disruption by interfering peptides on deSUMOylation dynamics, with mutations like R752K/R726K impairing stability and catalytic cooperativity. This study integrates *in silico* analyses with *in vivo* MCAO experiments to elucidate how CaMBD peptides modulate nNOS activity, SUMO1 modification, and SENP1-mediated deSUMOylation. These findings will clarify the competitive interplay between CaM allosteric activation and SUMO1 modification in nNOS hyperactivity, providing structural insights for designing targeted inhibitors to mitigate neurotoxicity in ischemic stroke and related disorders, ultimately advancing novel therapeutic strategies.

## Results

### Detection and analysis of the efficacy and cell membrane permeability of peptides in a mouse MCAO model

**FIGURE 1:**
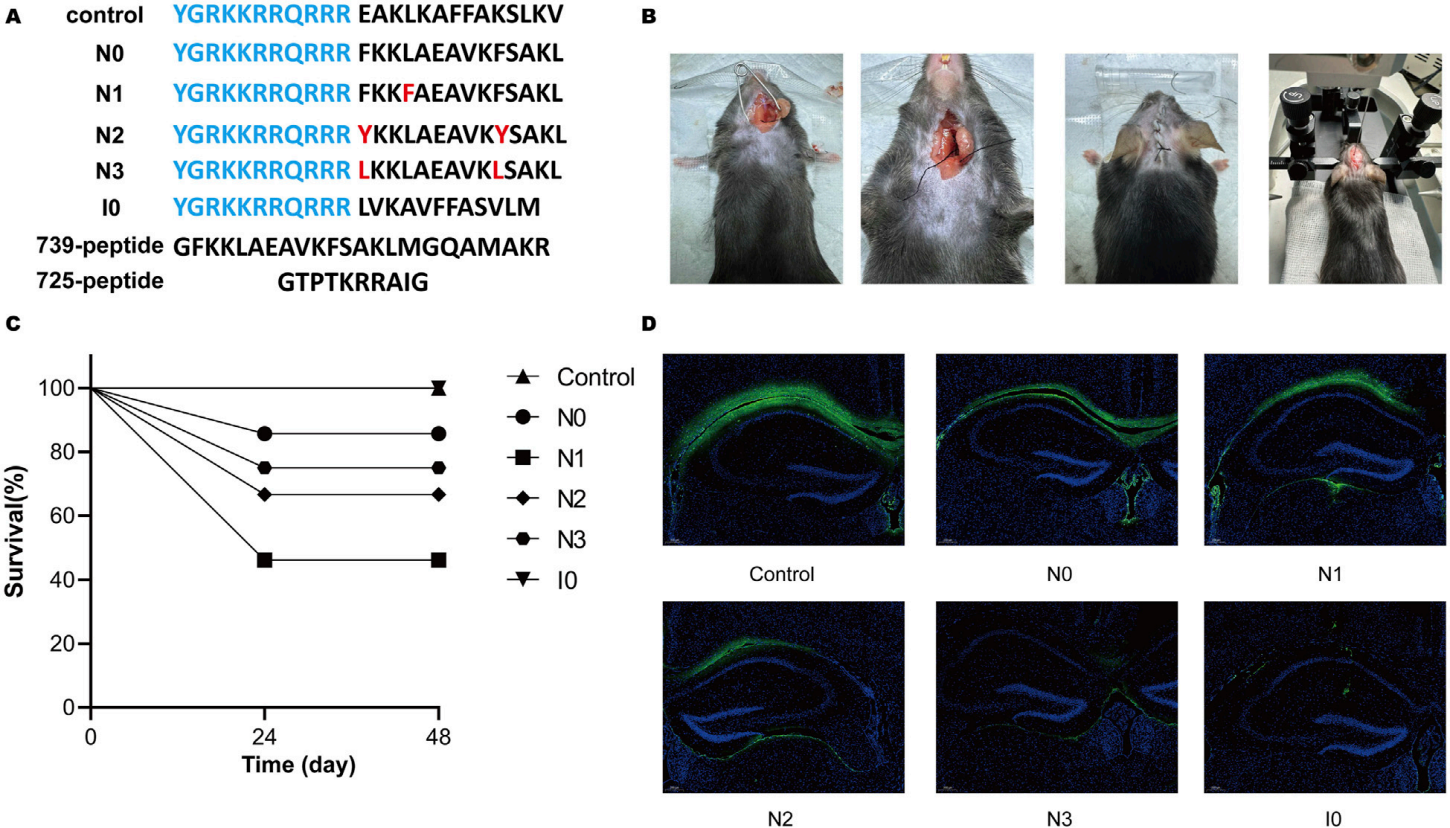
**(A)** Amino acid sequences of all peptides utilized in this study. Transmembrane sequences are highlighted in blue, and residues in red denote site-specific mutations relative to peptide N0. **(B)** The experimental platform, optimized for the mouse middle cerebral artery occlusion (MCAO) model, incorporates refined surgical procedures and instrumentation, including a stereotaxic injector. **(C)** Survival rates of mice were assessed 48 h post-injection following administration of 1 μL of solution into the cortical M1 and hippocampal CA1 regions. **(D)** Fluorescence microscopy (excitation: 494 nm, emission: 520 nm) was used to visualize FITC-labeled mouse brain sections, with imaging centered on the hippocampal CA1 region around the injection tract to evaluate the diffusion of interfering peptides within the hippocampus.

The following six interfering peptides were designed in this laboratory based on the mutation of the NOS-CaMBD domain in rats: nNOS-CaMBD polypeptide at positions 731–744 (N0), iNOS-CaMBD polypeptide at positions 515–526 (I0, serving as another control), control (Scrambled peptide group of nNOS-CaMBD), N1 (with L734F mutation in N0), N2 (with double mutations of F731Y and F740Y in N0), and N3 (with triple mutations of F731L, V738L, and F740L in N0). The effects on the mouse middle cerebral artery occlusion (MCAO) model were preliminarily verified (Figure 1A). We established a robust experimental platform and successfully developed a stable mouse MCAO model, laying a solid foundation for subsequent experiments (Figure 1B). Survival observations showed that N0, N3, and I0 peptide groups exhibited no significant impairment in survival, indicating no obvious toxicity under the established conditions, whereas the N1 and N2 groups showed marked declines in survival, suggesting differential toxicity (Figure 1C). Further, C57 mice were re-injected with equal peptide doses under identical conditions. Two hours later, brain tissues were harvested and sectioned. The tissues were then observed via fluorescence microscopy to assess diffusion of FITC-labeled peptides (excitation 494 nm, emission 520 nm) in hippocampal CA1 and membrane permeability to neurons (Figure 1D). Results showed better tissue spread in the control group, increases NO release in N0, N1, and N2, and poorer diffusion in N3 and I0 (worst in I0). Weak intracellular fluorescence signals indicated limited neuronal membrane permeability, warranting further validation. Collectively, these results suggest differential diffusion capacities of peptides in brain tissue. Notably, N1 and N2, despite relatively better diffusion than N3, showed less survival rate, potentially due to competitive binding to calmodulin (CaM). This hypothesis, based on current data, requires rigorous validation via Western blot analysis in hippocampal CA1 regions.

### The N0 peptide exhibits morphological but not significant behavioral neuroprotective effects in the MCAO model

**FIGURE 2:**
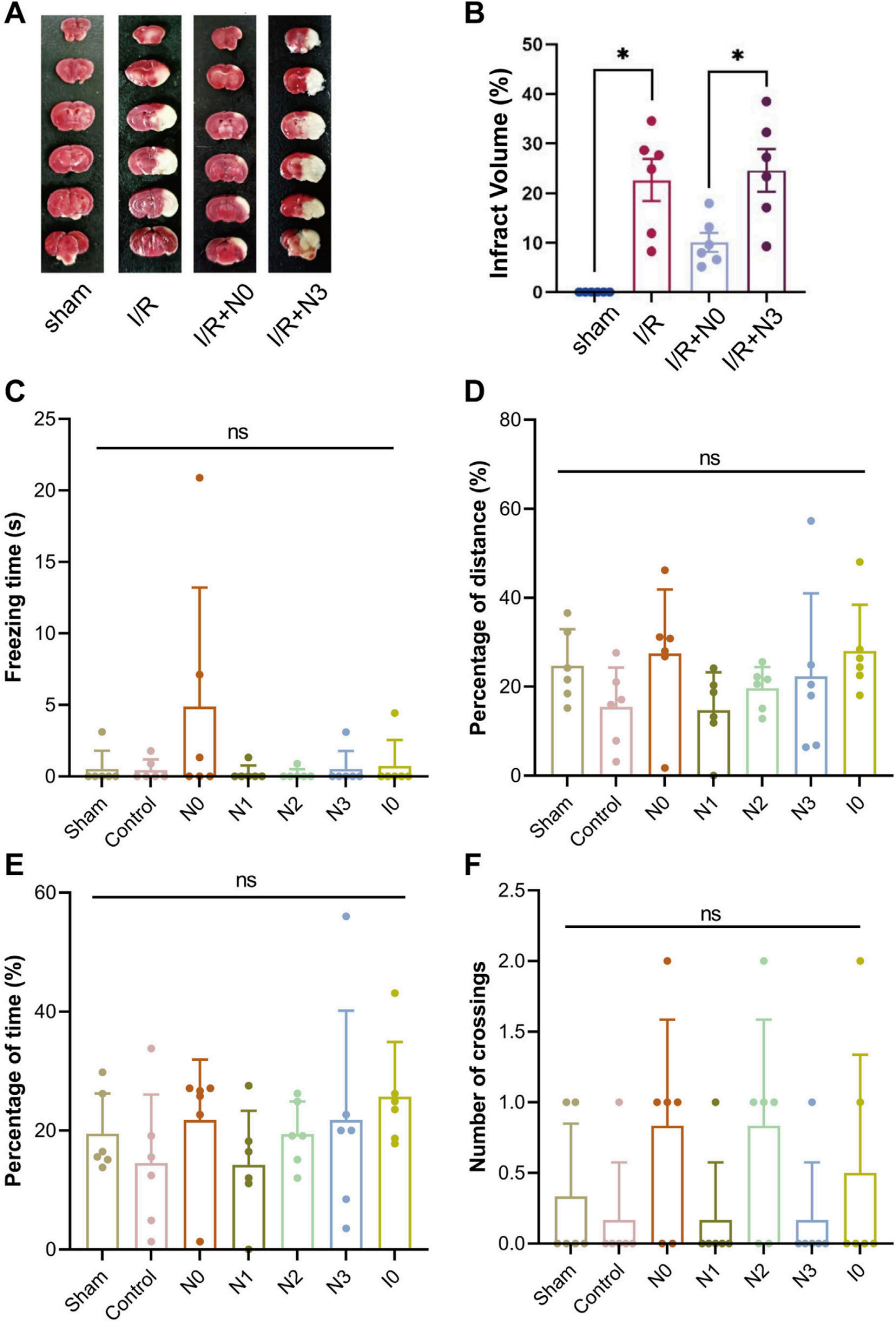
**(A)** Infarct volume in mouse brain tissue was quantified using 2,3,5-triphenyltetrazolium chloride (TTC) staining following the establishment of the middle cerebral artery occlusion (MCAO) model and injection of Peptide N0 or N3. **(B)** Statistical analysis of infarct volumes in mouse brain tissue. **(C–F)** Using a stereotaxic injector, 1 μL of each peptide was administered into the hippocampal CA1 and cortical M1 regions across 6 mouse groups. Two hours post-injection, MCAO was induced in all groups except the Sham group. On Day 3 post-MCAO, mice were subjected to the Morris Water Maze test. On Day 7, following platform removal, the following parameters were evaluated: **(C)** immobility time in the target quadrant, **(D)** total swimming distance, **(E)** active swimming time, and **(F)** number of platform crossings.

To further investigate the neuroprotective effects of peptides in mice with middle cerebral artery occlusion (MCAO), we selected CaMBD peptide (N0) and N3 peptide, which significantly increased NO release in cells, established the MCAO model, and administered N0 and N3 peptides via intracerebroventricular injection. Brain tissue damage was analyzed by 2,3,5-triphenyltetrazolium chloride (TTC) staining and compared with the sham operation group and ischemia-reperfusion control group (I/R group). The degree of brain tissue damage was observed via morphological observation (Figures 2A,B). Results showed that the cerebral infarction volume in the N0 peptide group was significantly reduced, while the N3 peptide group showed no significant change. To explore the effects of peptides on learning and memory functions, a Morris water maze test was conducted to record the movement trajectories of mice and analyze behavioral data on day 7 after platform removal, including residence time in the target quadrant, movement distance, duration, and platform crossing frequency (Figures 2C–F). No significant differences were observed in the movement trajectories or behavioral indices among peptide-injected groups. These findings indicate that N0 peptide exhibits morphological neuroprotective effects, but no significant behavioral neuroprotection. These findings, combined with previous neuronal results from our prior study mentioned above, indicate that the regulation of nNOS activity in the body is affected by multiple mechanisms. The design and improvement of interfering peptides need to consider the effects of CaM allosteric activation and SUMOylation modification on the nNOS-CaMBD region.

### Adequate binding between the structural region surrounding the SUMOylation site and SENP1 are the prerequisites for the deSUMOylation of nNOS peptide

**FIGURE 3:**
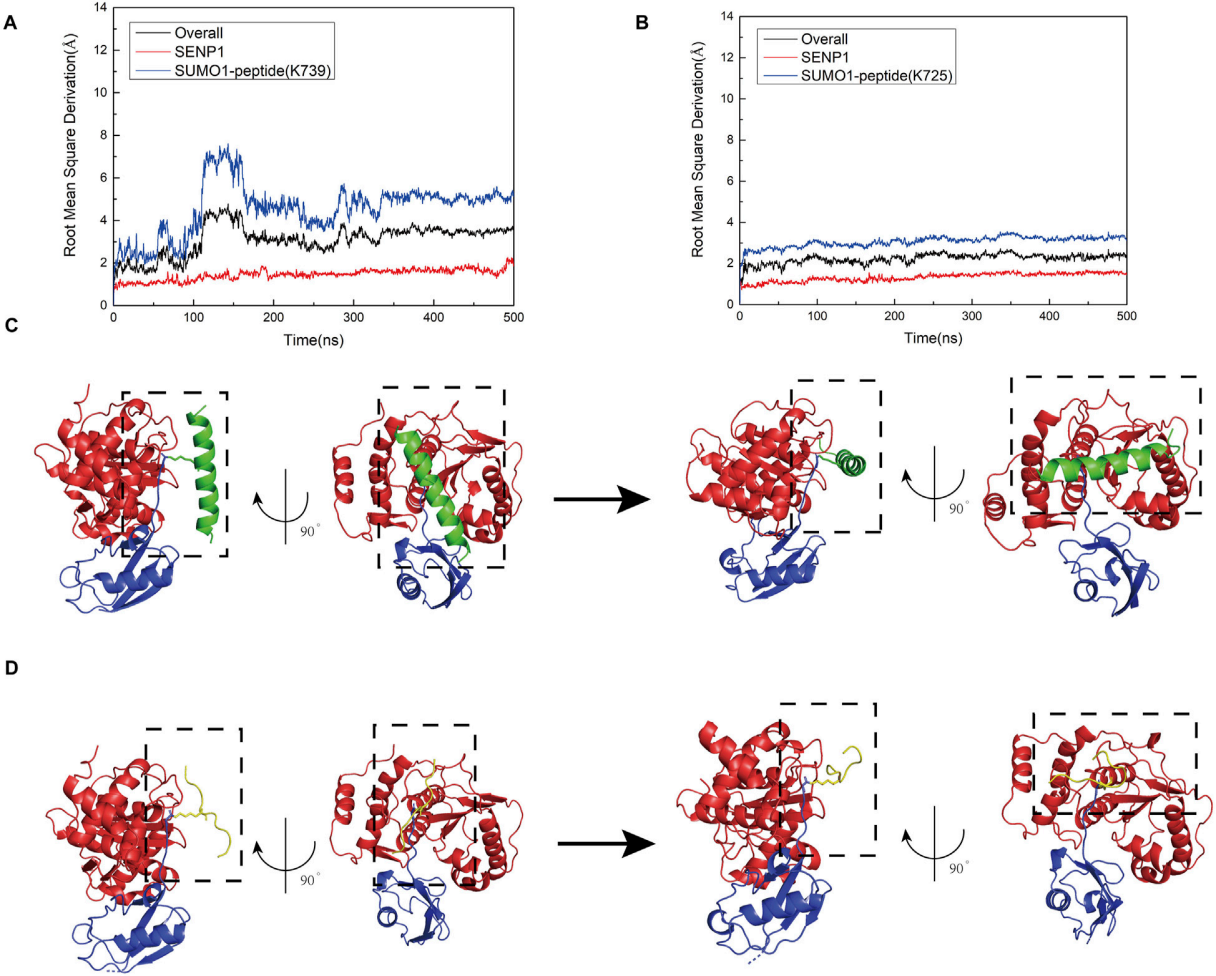
**(A)** Root mean square deviation (RMSD) plots showing the relationship between simulation time and the structural dynamics of the overall model (black curve), SENP1 (red curve), and SUMO1 peptide (blue curve) for the SENP1-SUMO1-peptide (K739) complex. **(B)** RMSD plots depicting the relationship between simulation time and the structural dynamics of the overall model (black curve), SENP1 (red curve), and SUMO1 peptide (blue curve) for the SENP1-SUMO1-peptide (K725) complex. **(C)** Visualizations of the structural evolution of the SENP1-SUMO1-peptide (K739) complex, showing the initial structure (first image) and the secondary structure at 500 ns (second image) during the molecular dynamics simulation. **(D)** Visualizations of the structural evolution of the SENP1-SUMO1-peptide (K725) complex, illustrating the initial structure (first image) and the secondary structure at 500 ns (second image) during the molecular dynamics simulation.

First, the nNOS K725 and nNOS K739 deSUMOylation models were built using molecular docking, and molecular dynamics simulations were used to verify the relative stability of the two models. Root-mean-square deviation (RMSD) calculations demonstrated that Models 1 and 2 reached a stable state after 200 ns (Figures 3A,B). The overall RMSD value for Model 1 remained below 5 Å, whereas that for Model 2 remained below 3 Å, indicating that the simulations accurately reflected the characteristics of the crystal structure. We found that during the simulation process, both Model 1 and Model 2 underwent a conformational change and finally reached an equilibrium state. We separately extracted the RMSD values for SENP1 and SUMO1 peptides from both models and found that the conformational changes in Models 1 and 2 were primarily due to the SUMO1 peptide. To observe the conformational changes in each part of the two models more clearly during the simulation process, we extracted Key conformations at time points from the RMSD graph are shown in Figures 3C,D. Comparison revealed that the main conformational changes in Models 1 and 2 occurred at the junction of SUMO1 and its adjacent structures like peptide, where the initially loose state gradually became more compact. As the models reached equilibrium, the contact between SENP1 and the SUMO1 peptide became more complete. Therefore, adequate binding between the structural regions surrounding the SUMOylation sites and SENP1 was concluded to be a prerequisite for the occurrence of deSUMOylation.

### Amino acid environment surrounding the K739 site of NOS was more favorable for the binding of SENP1 to the SUMO1-peptide than that surrounding the K725 site

**FIGURE 4:**
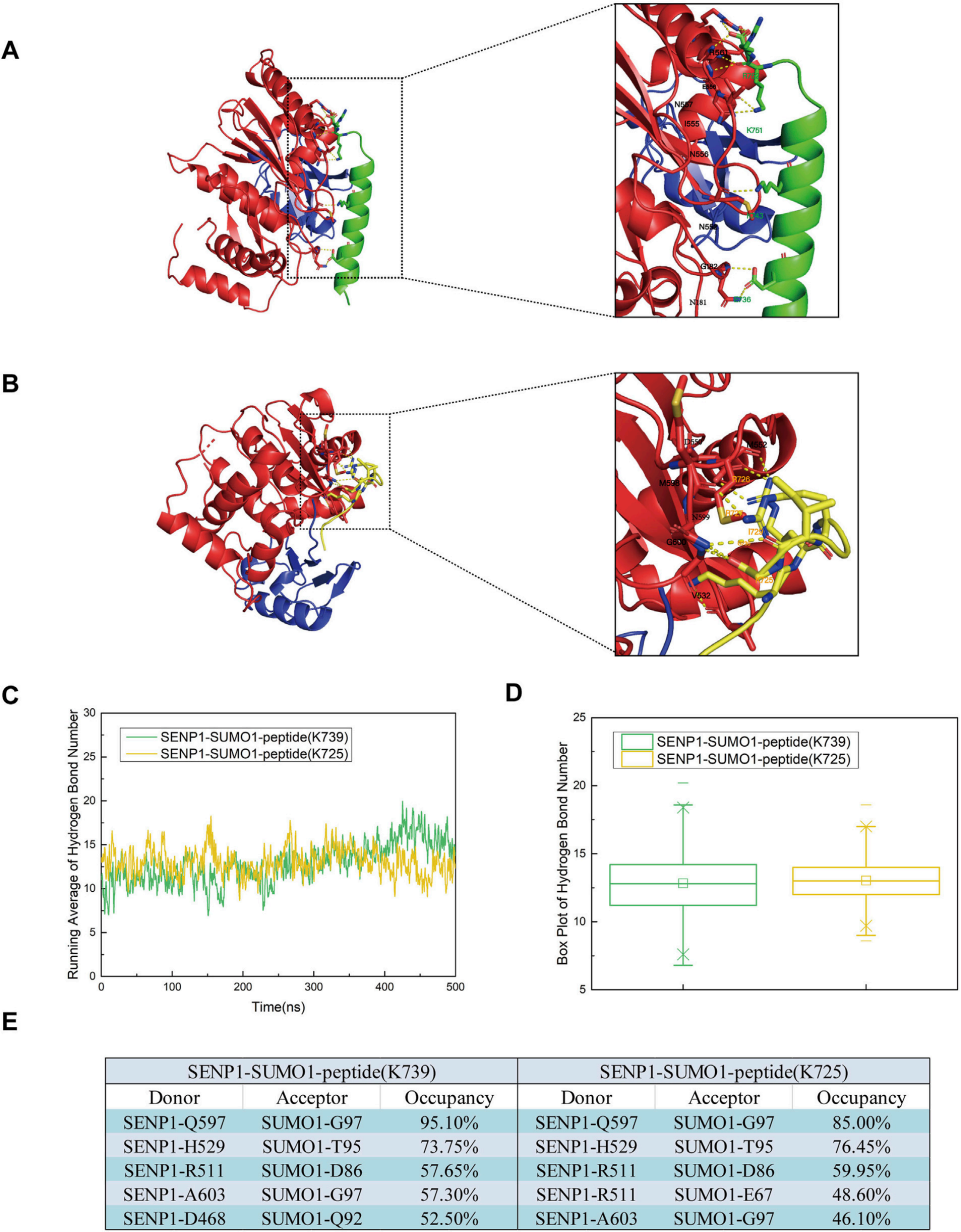
**(A)** Diagrams illustrating the secondary structures of the SENP1-SUMO1-peptide (K739) and SENP1-SUMO1-peptide (K725) complexes at 500 ns during the molecular dynamics simulation. **(B)** Representation of residues involved in polar contacts between SENP1 (red) and SUMO1-peptide (blue), with the K739 site (green) and K725 site (yellow) highlighted within dashed boxes for each respective model. **(C)** Time-dependent average number of hydrogen bonds in the SENP1-SUMO1-peptide (K739) and SENP1-SUMO1-peptide (K725) complexes. **(D)** Box plots depicting the distribution of hydrogen bond counts for each model, with quartile ranges indicated. **(E)** Identification of the top five hydrogen bonds in each model, specifying donor and acceptor residues.

To determine the key amino acid residues involved in the binding of SENP1 to the SUMO1 peptide, we analyzed the electrostatic interactions in the two models (Figures 4A,B). The simulation results revealed that in Model 1, residues N557 and R561 of SENP1, along with R752 of the peptide (K739), exhibited significant electrostatic interactions. Additionally, residues I555, N556, N557, and E558 of SENP1, together with K751 of the peptide (K739), also demonstrate strong electrostatic interactions. Furthermore, polar amino acid residues, including E736 and K743 of the nNOS peptide (K739), as well as M552, N599, and G600 in SENP1, were identified as playing a critical role in the binding of SENP1 to the SUMO1-nNOS (K739). In Model 2, residues V532 of SENP1 and R725 of the peptide (K725), as well as D550 and M552 of SENP1 and R726 of the peptide (K725), display notable electrostatic interactions. Specifically, V532 of SENP1 directly engages with the K725 residue of the peptide through electrostatic interactions. Moreover, polar amino acid residues such as R727, I729, and G730 of the peptide (K725), and M598, N599, and G600 of SENP1, may significantly contribute to the binding of SENP1 to the SUMO1-peptide (K725). Collectively, Model 1 demonstrated a higher frequency of robust electrostatic interactions between SENP1 and the nNOS peptide (K739), along with an increased presence of amino acid residues critical for the binding of SENP1 to the SUMO1-nNOS (K739). Consequently, these findings suggest that the amino acid environment surrounding the K739 site in Model 1 is more conducive to the binding of SENP1 to the SUMO1-peptide compared to the K725 site in Model 2.

In addition to electrostatic interactions, hydrogen bonds played a crucial role in protein interactions. To further investigate and analyze the changes in hydrogen bonding in the two models throughout the simulation process, we conducted hydrogen bonding statistics with a cutoff distance of 3.5 Å and a cutoff angle of 120°. The top five groups of residues paired with the average number of hydrogen bonds and high hydrogen bond occupancies are shown in Figures 4C–E. During the entire simulation process, the average number of hydrogen bonds in both models remained relatively stable and low. However, in Model 1 and Model 2, the average number of hydrogen bonds increased, fluctuated after 100 ns, and eventually stabilized. This suggested that Model 1 and Model 2 underwent conformational changes after 100 ns and reached a final equilibrium state. This further indicated that during the stable conformational process formed by the combination of SENP1 and SUMO1, hydrogen bonding promoted docking. Furthermore, our analysis revealed that the average number of hydrogen bonds in Model 1 was marginally higher than in Model 2 following equilibrium. Examination of the top five residue groups exhibiting high hydrogen bond occupancy rates (Figure 4E) indicated that hydrogen bonds predominantly formed between SENP1 and SUMO1 in both models, with minimal differences in the composition of these residue groups and their respective hydrogen bond occupancy rates between the two models. Notably, among the top five residue groups with high hydrogen bond occupancy in Model 1, one group involved interactions between SENP1 and the peptide (K739). Consequently, we conclude that hydrogen bonding is a critical factor in the deSUMOylation of both the K739 and K725 sites in SENP1-catalyzed nNOS. Moreover, the number and stability of hydrogen bonds between SUMO1 and SENP1 at the K739 site are greater than those at the K725 site in SENP1-catalyzed nNOS deSUMOylation.

### Residues of SENP1 in model 1 exhibited higher flexibility

**FIGURE 5:**
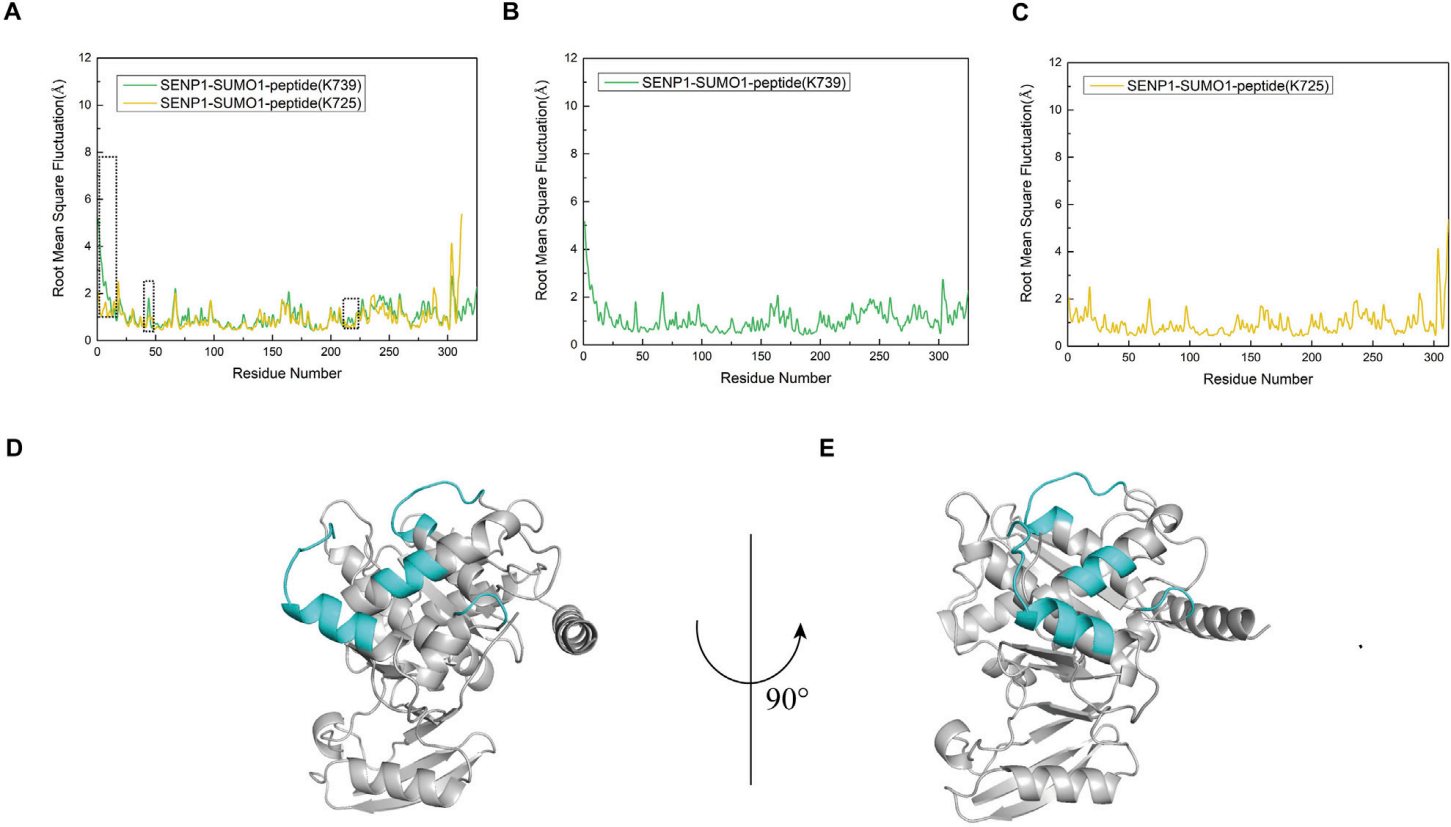
Root mean square fluctuation (RMSF) analysis of amino acid residues for each model, expressed in angstroms (Å). **(A)** RMSF profiles of the SENP1-SUMO1-peptide (K739) and SENP1-SUMO1-peptide (K725) complexes, depicted by green and yellow curves, respectively. A black dashed line highlights regions where residue flexibility in SENP1-SUMO1-peptide (K739) exceeds that of SENP1-SUMO1-peptide (K725). **(B)** RMSF plot for the SENP1-SUMO1-peptide (K739) complex. **(C)** RMSF plot for the SENP1-SUMO1-peptide (K725) complex. **(D,E)** Regions of elevated flexibility in SENP1-SUMO1-peptide (K739), highlighted in blue-green and corresponding to the boxed regions in **(A)**.

To investigate the flexibility of each component in the two models, we calculated the root mean square fluctuation (RMSF) values and conducted conformational analysis. Analysis of the Root Mean Square Fluctuation (RMSF) plots for Models 1 and 2 indicated that the overall RMSF values for both models were comparable, suggesting similar levels of flexibility across the amino acid residues in each model (Figures 5A–C). Nevertheless, specific regions of SENP1 in Model 1—namely E419-K432, N461-L463, C613-N621, and K632-W636—exhibited slightly elevated flexibility compared to Model 2, although the differences were not statistically significant (Figures 5D,E). These findings suggest that certain flexible regions of SENP1 in Model 1 may accommodate a broader range of conformational states. Based on these findings, we hypothesized that SENP1 is more likely to bind to the SUMO1 K739 peptide and effectively facilitate deSUMOylation.

### Correlation coefficient analysis revealed a robust correlation between SUMO1-peptide (K739) and SENP1

**FIGURE 6:**
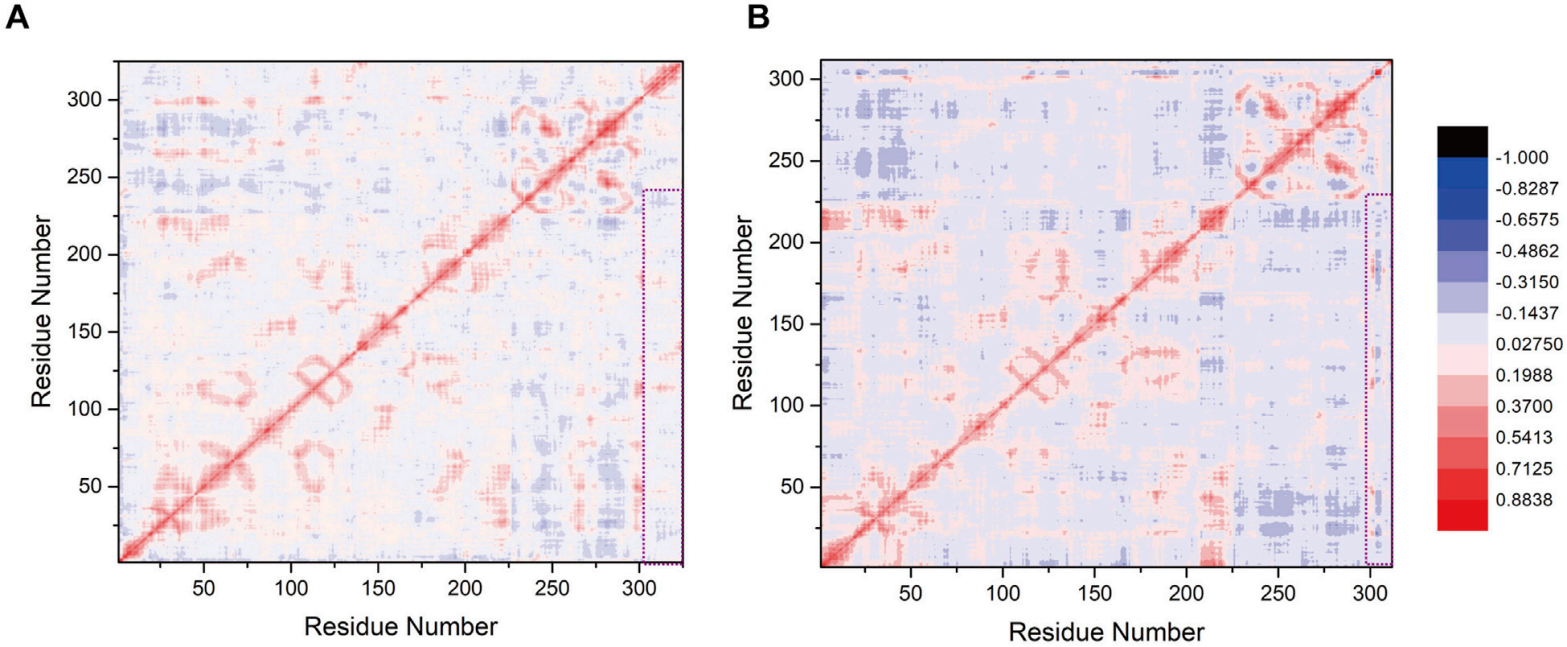
**(A)** Correlation factor analysis for 325 residues in the SENP1-SUMO1-peptide (K739) complex. **(B)** Correlation factor analysis for 312 residues in the SENP1-SUMO1-peptide (K725) complex. Highly correlated residues are depicted in red, while anti-correlated residues are shown in blue, with the legend provided on the right side of **(B)**. The purple box highlights the correlation analysis results between SENP1 and the two models.

To assess the influence of K739 and K725 site selection on the internal correlation between SENP1 and SUMO1 peptides, we computed inter-residue correlation coefficients reflecting the vibrational characteristics of each amino acid residue in the SENP1 and SUMO1 peptides. This analysis aimed to identify the correlations between amino acid fragments and their populations in both models. Initially, we observed that minor differences in site selection between the two models led to similar correlation coefficient graphs. However, the distribution of correlation factors exhibited slight variations (Figures 6A,B). Specifically, the highlighted segments in purple boxes in Model 1 (numbered 20–226, 290–302, and 303–325 corresponding to G438-L644 of SENP1, E85-G97 of SUMO1, and G730-R752 residues of the K739 peptide) showed positive values and positive correlations. The amino acid residues exhibited mutual attraction, suggesting that the presence of the peptide (K739) enhanced the cohesiveness of SENP1’s interaction with SUMO1, leading to a more compact conformational state. In contrast, in Model 2, the purple box sections (numbered 20–226, 290–302, and 303–312 corresponding to G438-L644 of SENP1, E85-G97 of SUMO1, and G721-G730 residues of the peptide (K725)) exhibited near-neutral correlations with minimal negative values. This suggested that the residue groups experienced negligible global correlation or anticorrelation changes. Therefore, we concluded that selecting the K739 site facilitated SENP1’s binding to the SUMO1 K739 peptide and accordingly enhanced its catalytic effects.

### Free energy calculations provided additional insights into the selectivity of SENP1 binding to the two SUMOylation sites of nNOS

**FIGURE 7:**
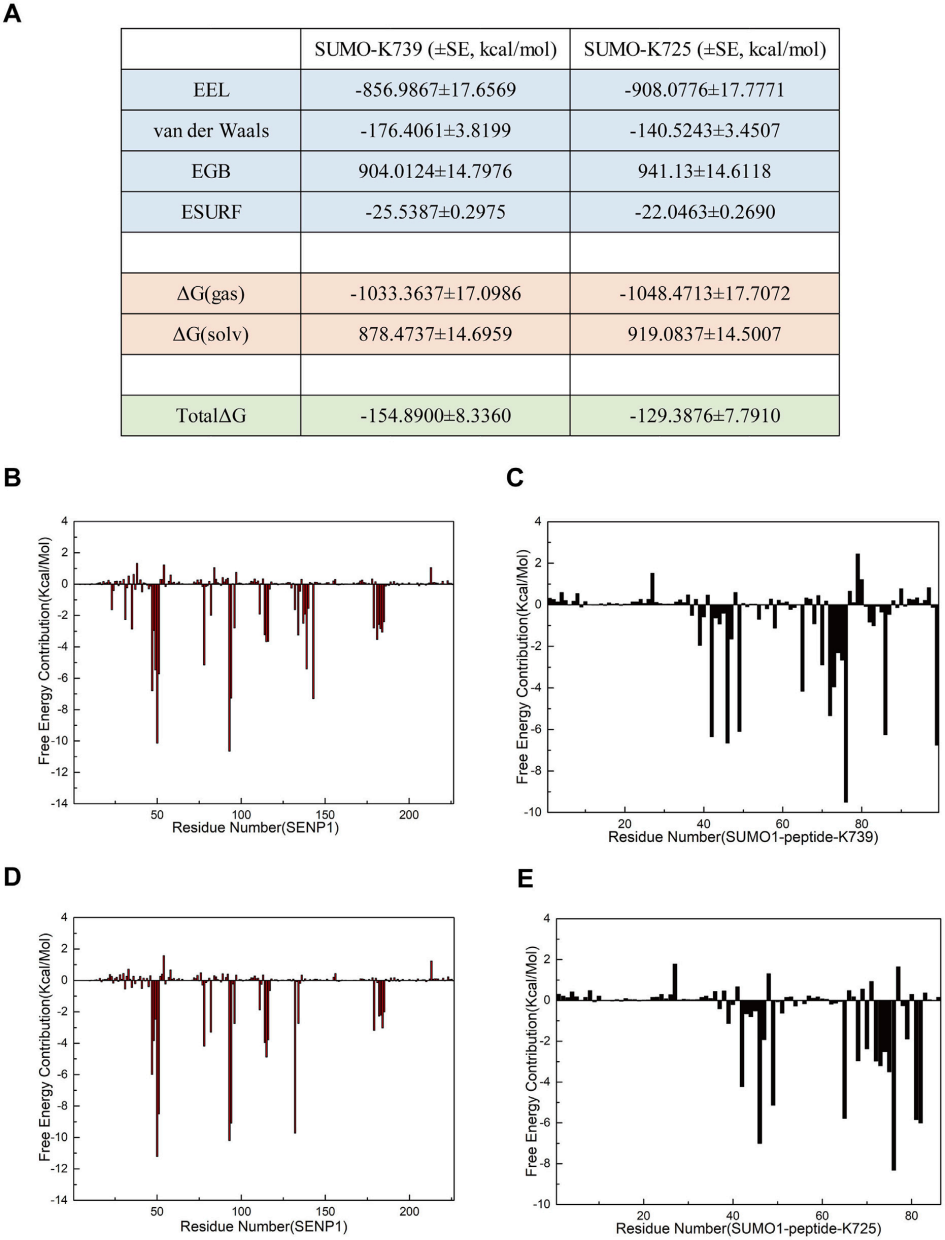
Free energy calculations and decomposition for each model. **(A)** Components of the free energy calculation, expressed in kcal/mol, with standard error (SE) included (the free energy calculations were performed using 96 frames of data, such that the standard deviation is equal to the standard error multiplied by the square root of 96). **(B,C)** Energy decomposition diagrams for the receptor component SENP1 and the ligand component SUMO1 K739 peptide in the SENP1-SUMO1-peptide (K739) complex, respectively. **(D,E)** Energy decomposition diagrams for the receptor component SENP1 and the ligand component SUMO1 K725 peptide in the SENP1-SUMO1-peptide (K725) complex, respectively.

Analysis of electrostatic interactions, hydrogen bonding statistics, and correlation coefficients provided a comprehensive overview of the roles played by individual amino acid residues or groups in the SUMO1 peptide/SENP1 interaction at the K739 and K725 sites. However, certain key amino acid residues that influence the SUMO1 peptide/SENP1 interaction remain elusive, since these calculations only considered partial amino acid residue characteristics. Additionally, critical interactions, such as van der Waals forces, could be explored further through free energy calculations. To delve deeper into the selectivity of SENP1 for binding to the two SUMOylation sites of nNOS and to elucidate the underlying molecular relationships, we conducted binding free energy predictions in implicit solvents (Figure 7A). We selected the last stable 50 ns (200 frames) from the 500-ns simulations for the free energy calculations. The SUMO1 peptide served as the ligand and SENP1 served as the receptor in these calculations. The results indicated the total energy of Model 1 is more negative compared to that of Model 2, suggesting that the binding of the SUMO1 K739 peptide with SENP1 was energetically more favorable. In both the models, electrostatic interactions (EEL) and van der Waals interactions (vdW) contributed to the binding of the SUMO1 peptide to SENP1, with EEL contributing more substantially to the overall free energy than vdW. This indicated that the interactions between SUMO1 peptide and SENP1 primarily relied on polar contacts. Notably, Model 1 exhibited a larger vdW, indicating stronger contacts, which favored SENP1’s selection of the K739 site. The free energy decomposition results highlighted the importance of the residues involved in polar contacts/hydrogen bonds and those facilitating van der Waals interactions in the SENP1 site selection process.

To identify additional key residues involved in the interaction between SENP1 and SUMO1 in both the models, we decomposed the binding free energy results into individual residues (Figures 7B–E). Subsequent analysis revealed the critical residues that contributed significantly to the binding free energy in each model. The results indicate that in Model 1, the energy-critical residues contributing more than 5.0 kcal/mol to the binding free energy include M552 and Q597 of SENP1, and R752 of the peptide (K739), consistent with the findings from the electrostatic interaction analysis. Additionally, Model 1 identified previously unrecognized key residues, namely W465, D468, R511, and W512 of SENP1, as well as R63, E67, and G97 of SUMO1, and K739 of the peptide (K739), each contributing more than 6.0 kcal/mol to the binding free energy. Notably, the SUMOylation site K739 contributes a binding free energy of >6.3 kcal/mol. In contrast, Model 2 identified energy-critical residues, including D550 of SENP1, and R726 and K725 of the peptide (K725), each contributing over 5.5 kcal/mol to the binding free energy, aligning with the electrostatic interaction analysis. Among these, the SUMOylation site K725 contributes a binding free energy of >5.5 kcal/mol. Model 2 also revealed some unnoticed key residues, such as D468, E469, R511, and W512 in SENP1; E67 and G97 in SUMO1, each contributing over 6.0 kcal/mol to the binding free energy. Interestingly, D468, R511, and W512 in SENP1; E67 and G97 in SUMO1 were identified as crucial residues in both the models. Moreover, a greater number of residues contributed more than 5.0 kcal/mol of binding free energy in Model 1 than in Model 2. The regions R522-R561 and Q597-A603 in SENP1,and the entire region of SUMO1 exhibited higher binding free energies in Model 1 than in Model 2, contributing to the overall higher free energy of Model 1. These findings indicated that the amino acid residue structure of the SUMO1 K739 peptide facilitated a more favorable binding interaction with SENP1 in Model 1, suggesting the amino acid environment of Model 1 to be more conducive to the binding of SENP1 to SUMO1 peptide.

### The R752K mutation of the peptide in model 1 weakens the stable binding and interaction network between SUMO1-peptide and SENP1

**FIGURE 8:**
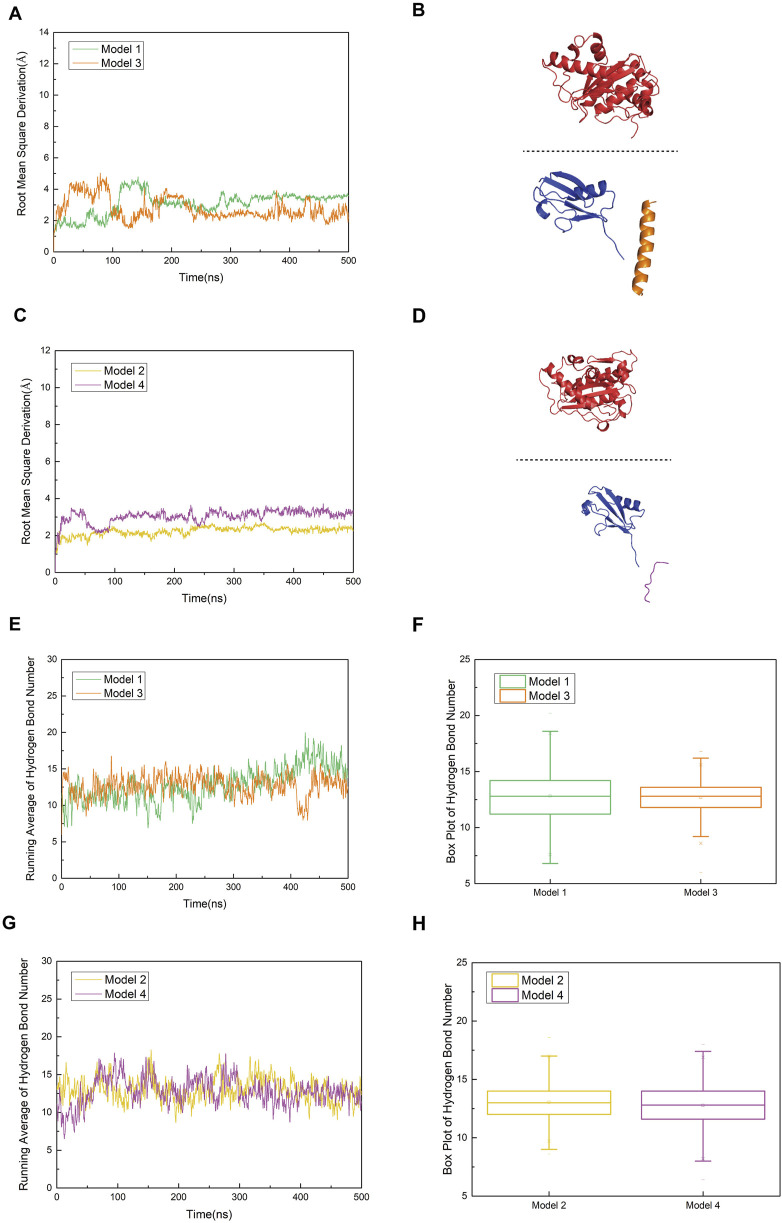
**(A)** Comparison of root mean square deviation (RMSD) curves for Model 1 (original SENP1-SUMO1-peptide (K739)) and Model 3 (SENP1-SUMO1-peptide (K739) with R752K mutation), represented by green and orange curves, respectively. **(B)** Secondary structure diagram of Model 3 at 500 ns during the molecular dynamics simulation, to clearly illustrate the conformational changes of the SENP1–SUMO–peptide complex and the variations in their relative positions, the SENP1 structural region is displayed using box-boundary mirroring. **(C)** Comparison of RMSD curves for Model 2 (original SENP1-SUMO1-peptide (K725)) and Model 4 (SENP1-SUMO1-peptide (K725) with R726K mutation), depicted by yellow and purple curves, respectively. **(D)** Secondary structure diagrams of Model 3 and Model 4 at 500 ns during the molecular dynamics simulation (SENP1 is same as in Figure 8B). **(E,G)** Statistical analysis of average hydrogen bond counts for Model 1 versus Model 3 and Model 2 versus Model 4, plotted against simulation time. **(F,H)** Box plot analysis of hydrogen bond counts across the four models, with the box range indicating the 25th and 75th percentiles.

As shown in Figures 8A,C, the trends of the RMSD curves reflect differences in the structural stability of the complexes before and after mutation. Model 1 (with R752) exhibited fluctuations between 2.0 and 5.0 Å throughout the simulation, indicating structural stability and suggesting that the binding between SUMO1-peptide (K739) and SENP1 is relatively stable. 1n contrast, Model 3 (R752K mutation) showed more pronounced RMSD fluctuations, indicating increased instability of the complex structure post-mutation, which may imply a gradual loosening of the complex. A similar pattern was observed in Model 4 (R726K mutation), which displayed higher RMSD values and greater structural perturbations compared to Model 2, suggesting that this mutation also weakens the structural coordination between SUMO1-peptide and SENP1 and the overall stability of the complex. These results collectively indicate that the complexes of both mutant peptides with SENP1 are more prone to structural loosening. Figures 8B,D depict the conformational states of Models 3 and 4 at 500 ns of molecular dynamics simulation. Notably, significant spatial separation was observed at the interface between SENP1 and SUMO1-peptide in the mutant models. The SUMO1-peptide domain transitioned from an initially embedded state in Models 1 and 2 to a partially detached configuration in the mutants. This suggests that the mutant peptides lose the spatial compatibility required to maintain a stable binding interface. Furthermore, this conformational drift may disrupt the spatial alignment between the catalytic center of SENP1 and the SUMOylated lysine residue, potentially leading to reduced enzymatic activity.

Hydrogen bond analysis (Figures 8E,F) revealed that Model 1 stably formed 5–8 hydrogen bonds with SENP1. As the molecular dynamics simulation progressed toward equilibrium, increasingly robust polar interactions were established between SENP1 and the peptide, indicative of a mutual stabilization mechanism within the ligand-enzyme complex. These results suggest that residue R752 in the wild-type peptide not only contributes an electrostatic interaction node but may also orchestrate a sophisticated hydrogen bond network, thereby maintaining the structural integrity of the binding interface. Although Model 3 (R752K mutant) initially exhibited a comparable hydrogen bond count to Model 1, a discernible divergence emerged after 400 ns, with Model 3 maintaining fewer hydrogen bonds at equilibrium. This discrepancy implies that while the R752K substitution preserves the side-chain positive charge—potentially sustaining electrostatic interactions with negatively charged SENP1 residues—the replacement of arginine’s extended guanidinium moiety, capable of multi-directional hydrogen bonding and π-stacking interactions, with the structurally simpler lysine disrupts the precise spatial alignment required for optimal complex stability. These observations underscore that despite isoelectric properties, the mutant residue confers diminished functional capacity. Inter-model comparison between Models 3 and 4 (Figures 8G,H) revealed subtler distinctions in hydrogen bonding patterns. Although mean hydrogen bond counts differed marginally, Model 4 consistently exhibited reduced hydrogen bond frequency and increased variability (as reflected by box plot statistics), suggesting that the R726K mutation similarly weakens polar interactions at the peptide-enzyme interface. However, given the inherently weaker electrostatic and hydrogen bonding landscape in the parental Model 2, the post-mutation decline in hydrogen bonding was less pronounced compared to the Model 1→3 transition. Structurally, the R752 residue is strategically positioned proximal to the SUMOylated K739 site, within a microenvironment rich in charged and polar residues (e.g., K751, E736). These residues collectively form an extensive polar network with SENP1 counterparts (e.g., N557, E558, R561), suggesting a critical scaffolding role for R752 in bridging the K739 substrate and the catalytic pocket of SENP1. Mechanistically, this architectural arrangement likely facilitates substrate positioning, transition state stabilization, and potentially allosteric modulation of the enzymatic active site during deSUMOylation. Disruption of this bridging function through mutagenesis abrogates efficient substrate docking, thereby compromising catalytic efficiency. Collectively, these findings implicate R752 as a linchpin residue in the molecular recognition and processing of the nNOS K739 site by SENP1, with its substitution by lysine profoundly undermining this essential regulatory mechanism.

### R726K mutation in the peptide of model 2 slightly alter the flexibility patterns of key residues

**FIGURE 9:**
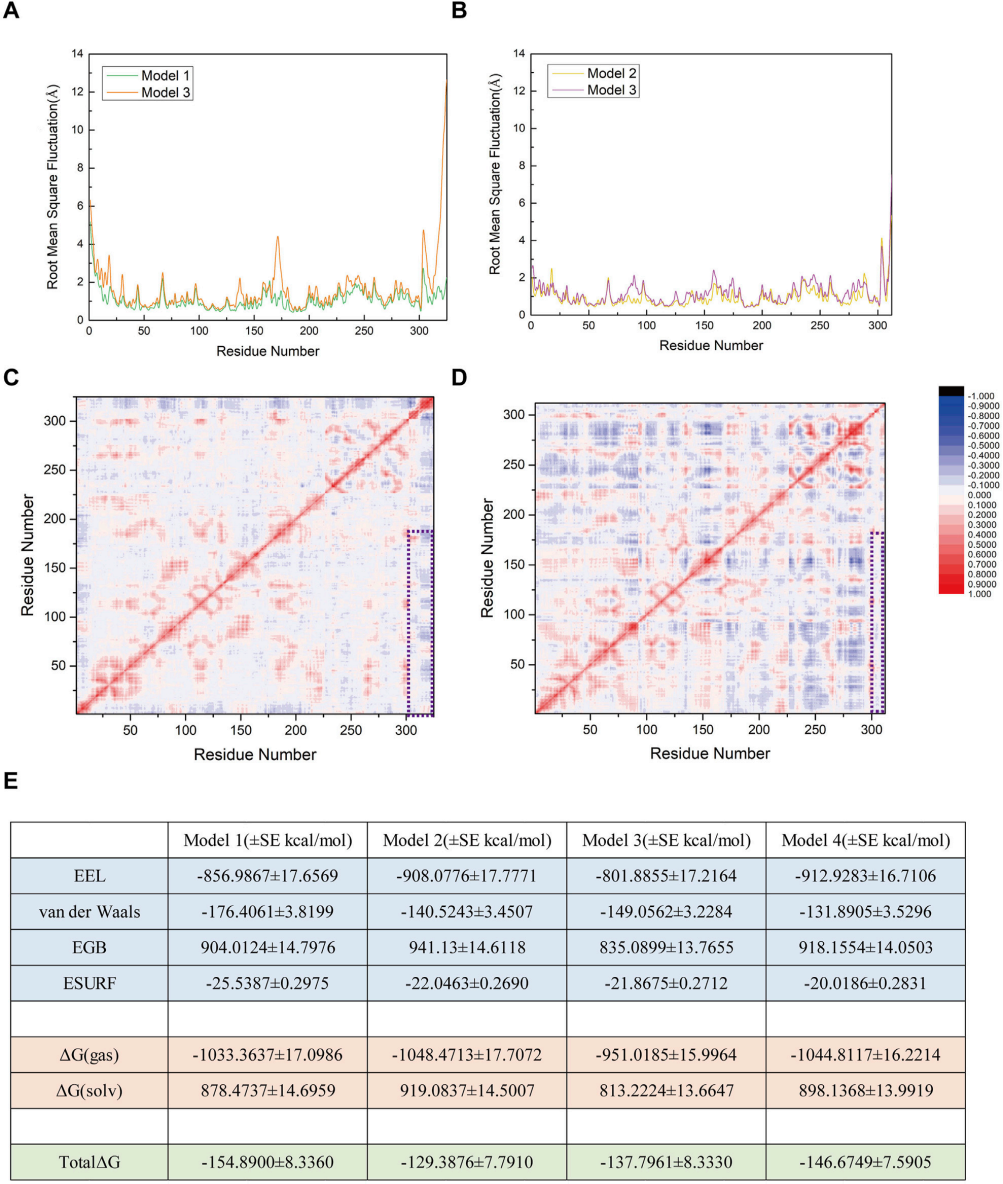
**(A)** Comparison of root mean square fluctuation (RMSF) values between Model 1 (original SENP1-SUMO1-peptide (K739)) and Model 3 (SENP1-SUMO1-peptide (K739) with R752K mutation), with RMSF values (Å) represented by green and orange curves, respectively. **(B)** Comparison of RMSF values between Model 2 (original SENP1-SUMO1-peptide (K725)) and Model 4 (SENP1-SUMO1-peptide (K725) with R726K mutation), with RMSF values (Å) depicted by yellow and purple curves, respectively. **(C)** Correlation factor analysis for 325 residues in Model 3. **(D)** Correlation factor analysis for 312 residues in Model 4. Highly correlated residues are indicated in red, and anti-correlated residues in blue, with the legend displayed to the right of **(D)**. The purple box highlights the correlation factor analysis results between SENP1 and the peptides in both models. **(E)** Free energy components for the four models, expressed in kilocalories per mole (kcal/mol), with standard error (SE) included (the free energy calculations were performed using 96 frames of data, such that the standard deviation is equal to the standard error multiplied by the square root of 96).

RMSF analysis (Figures 9A,B) revealed subtle yet significant increases in residue flexibility across Models 3 (R752K) and 4 (R726K) compared to Models 1 and 2. Elevated RMSF values localized to SENP1 regions suggest mutation-induced conformational destabilization, potentially reflecting intramolecular loosening or structural decoupling. In contrast, the lower RMSF profiles of Models 1 and 2 indicate greater conformational stability, critical for substrate recognition, catalytic positioning, and binding affinity. These findings suggest that mutations disrupt optimal SUMO1-peptide/SENP1 docking, thereby impairing deSUMOylation efficiency.

Residue fluctuation correlation heatmaps (Figures 9C,D) identified positive correlation regions (red) between SENP1 (G438-L644) and peptide segments (G730-R752 in Model 1; G721-G730 in Model 2), suggesting cooperative catalytic adjustments critical for substrate recognition. These correlations were abolished in Models 3/4 (R752K/R726K), particularly evident in Model 3 where adjacent peptide residues lost dynamic coupling with SENP1’s core (blue/gray regions). This indicates R752/R726 residues maintain both structural and dynamic enzyme-substrate. Free energy analysis (Figure 9E) showed Model 1’s binding energy (−154.89 kcal/mol) increased by ∼17 kcal/mol in Model 3, reflecting disrupted electrostatic/hydrogen bonding interactions. Conversely, Model 4 (R726K) exhibited lower free energy (−146.67 vs. −128.39 kcal/mol in Model 2), yet impaired stability and cooperativity, suggesting non-specific thermodynamic compensation rather than enhanced binding. Collectively, R752 acts as a critical polar hub near K739, with mutation-induced disruptions spanning structural, dynamic, and thermodynamic levels. While R726K preserves binding energy, it compromises catalytic efficiency via reduced flexibility and coupling. Both residues are indispensable for SENP1-mediated deSUMOylation.

### N1/N2/N3 peptides exhibit limited impact on the dynamic coupling and thermodynamic stability of SENP1-Mediated nNOS DeSUMOylation

**FIGURE 10:**
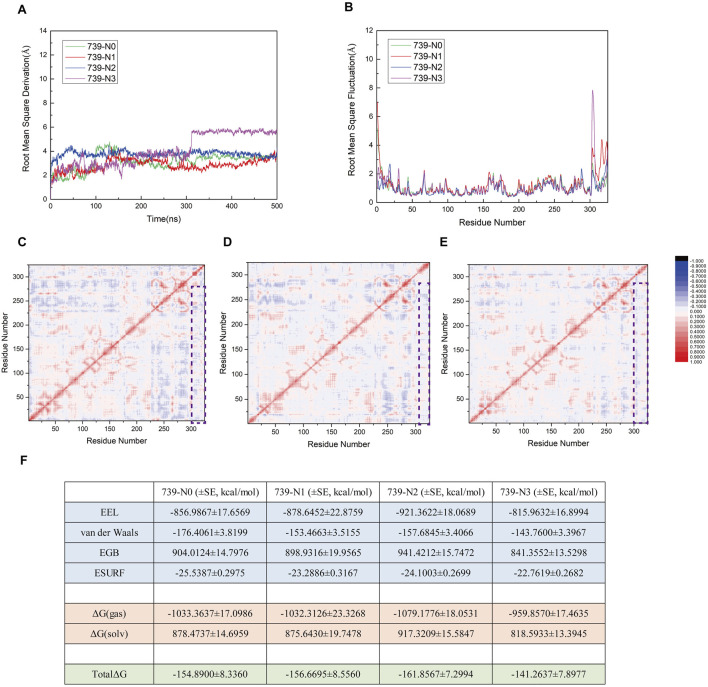
**(A)** Root Mean Square Deviation (RMSD) trajectories for the four models (739-N0, 739-N1, 739-N2, and 739-N3) over the duration of the simulation. **(B)** Comparative analysis of Root Mean Square Fluctuation (RMSF) profiles across the four models. **(C–E)** Residue–residue correlation factor maps for the 739-N1, 739-N2, and 739-N3 models, respectively. Positively correlated residues are depicted in red, while negatively correlated residues are shown in blue. A legend is provided to the right of panel **(E)**. Regions enclosed in purple boxes highlight the outcomes of correlation factor analysis between SENP1 and the peptide in the respective models. **(F)** Components of the calculated binding free energy for the four models, accompanied by their associated standard errors, expressed in kcal/mol.

To investigate the effects of interfering peptides on the SENP1-mediated deSUMOylation process of nNOS, we performed molecular dynamics (MD) simulation analyses on the following models: the original model 739-N0 and three complex models (739-N1, 739-N2, and 739-N3) in which the peptide was substituted with interfering peptides. The structural stability and RMSD results of these four models over a 500 ns simulation period are presented in Figure 10A; RMSF results are presented in Figure 10B. The RMSD curves indicate that all models achieved a stable state after approximately 300 ns, with fluctuations consistently maintained within the 2.0–6.0 Å range, suggesting that all four systems reached equilibrium during the simulation. Notably, the RMSD curves of the interfering peptide models 739-N1 and 739-N2 exhibited minimal deviation from the original model 739-N0, indicating that the introduction of interfering peptides N1 and N2 did not induce significant structural perturbations in the complexes. These models displayed tightly organized structural arrangements with no evident signs of dissociation or displacement, and the binding interface maintained a high degree of compatibility. The residue dynamic correlation results for the interfering peptide models 739-N1, 739-N2, and 739-N3 are shown in Figures 10C–E. All models displayed cooperative vibrational patterns similar to those of the original model 739-N0. Although the correlation coefficients were slightly reduced compared to the original model, the dynamic coupling characteristics were preserved. Notably, the 739-N2 model exhibited pronounced red correlation in key regions, indicating that its vibrational coordination was the most similar to the original model. In contrast, models 739-N1 and 739-N3 showed minor anti-correlated regions at the periphery, but no systemic decoupling trends were observed, suggesting that the cooperative motion between SENP1 and SUMO1-peptide was not disrupted by the presence of interfering peptides.

To further explore the thermodynamic effects of interfering peptides on the SUMOylation process of nNOS, MM/GBSA free energy calculations were systematically analyzed (Figure 10F). The free energy calculations revealed that the total binding free energy of the original model 739-N0 was −154.89 kcal/mol, indicating a strong thermodynamic driving force for binding. The binding free energies for models 739-N1, 739-N2, and 739-N3 were −156.6695, −161.8567, and −141.2637 kcal/mol, respectively, with differences within a range of 10–15 kcal/mol. Considering the standard error, these differences were deemed insignificant. In all models, electrostatic interactions (EEL) were the primary contributors to binding energy, followed by van der Waals interactions, while solvation effects remained largely unaffected by the introduction of interfering peptides, indicating that the polarity characteristics and molecular embedding at the critical binding interface remained stable. Notably, although the binding free energy of the 739-N3 model was slightly higher than that of the other models, its residue correlation remained largely unchanged, suggesting that the interfering peptide caused minor perturbations but was insufficient to disrupt the overall functional coupling network or fully impair the binding of SUMO1-peptide or the enzymatic conformation of SENP1.

In conclusion, within the current simulation scale and model systems, the interfering peptides exhibited limited effects on the dynamic coupling and thermodynamic stability of the deSUMOylation process, with no significant inhibitory or promotional trends observed. These findings suggest that the interfering peptides have not established a sufficient “docking competition mechanism” at the molecular level to disrupt the SENP1-nNOS interaction. Further validation of their mechanistic roles may require additional cellular or *in vivo* experiments.

## Discussion

Building on the previous findings of our laboratory, behavioral data suggest that the interfering peptides may activate additional regulatory mechanisms of nNOS by competitively binding to CaM, thereby promoting nNOS hyperactivation and increasing NO release (Wan and Wang, 2024). In our previous studies, we found that interference peptides designed based on the CaMBD of nNOS (namely N0, N1, N2, and N3) exhibited differential regulatory effects on NO production at the cellular level. Among them, N2 and N3 significantly increased NO release, while N0 had no notable effect. However, in the mouse MCAO model, a contrasting outcome was observed: administration of the N0 peptide alleviated ischemia-induced brain tissue damage, whereas N2 and N3 showed no such protective effects. This discrepancy was unexpected. Moreover, none of the peptides demonstrated significant improvement in cognitive or behavioral performance in the Morris water maze test, suggesting a lack of behavioral-level neuroprotection. Integrating these experimental findings with our previous molecular dynamics simulations of peptide–CaM interactions, we propose that the N0 peptide, although not binding to CaM as tightly as its mutants N2 or N3, retains a moderate affinity that allows for a balanced interaction. This moderate binding may prevent excessive competition with endogenous calmodulin, thereby avoiding excessive exposure of the CaMBD region on nNOS. Overexposure of the CaMBD, particularly at the K725 and K739 sites (with K739 being more critical), may facilitate aberrant SUMOylation, which has been associated with detrimental effects on neuronal viability. These results suggest that peptides designed based on the CaMBD of nNOS must take into account not only competitive interactions with CaM but also the impact on other regulatory mechanisms, such as SUMOylation. Achieving a balanced modulation of nNOS activity—neither excessive activation nor over-suppression—is likely essential for realizing effective neuroprotection.

SUMOylation is a pivotal posttranslational modification that regulates protein activity, stability, cellular localization, and signaling pathways. Alterations in SUMOylation of proteins play crucial roles in the regulation of neuronal and synaptic functions (Henley et al., 2018; Henley et al., 2021). Specifically, SUMO1 can bind to nNOS, inducing SUMOylation, thereby sustaining high nNOS activity and the continual release of excessive nitric oxide (NO) into neurons. The excess NO can upregulate pro-apoptotic factors in neurons, triggering neuronal apoptosis and other forms of nerve damage. The CaMBD of nNOS harbors two SUMOylation sites, K725 and K739, the latter being a predicted SUMOylation site according to tools like GPS-SUMO. To strategically inhibit nNOS activity, we investigated the potential sequences of SENP1-mediated deSUMOylation modifications at these two sites. Therefore, we generated two binding models for SENP1/SUMO1 peptide interactions based on variations in SENP1 site selection. We conducted comprehensive analyses, including RMSD, polar contact assessment, hydrogen bonding statistics, RMSF, correlation coefficient evaluation, and free energy calculations. The models offered stable and detailed insights into the structural interactions between SENP1 and SUMO1 peptide (nNOS), thereby enhancing our understanding of nNOS deSUMOylation. Furthermore, these findings provided insight into the design and refinement of specific inhibitors targeting nNOS SUMOylation, potentially paving the way for novel therapies to treat neurological and psychiatric disorders associated with excessive nNOS activation.

We observed that adequate binding of the structure surrounding the SUMOylation site to SENP1 was essential for initiating deSUMOylation. Free energy decomposition highlighted the significant role of electrostatic interaction energy (EEL) in mediating the binding between SUMO1 peptide and SENP1, emphasizing its importance in the interaction. Electrostatic interaction analysis revealed that in Model 1, residues N557 and R561 of SENP1, along with R752 of the peptide (K739), as well as residues I555, N556, N557, and E558 of SENP1, together with K751 of the peptide (K739), exhibit robust electrostatic interactions. Furthermore, several polar amino acid residues, including E736 and K743 of the peptide (K739), and M552, N599, and G600 of SENP1, likely play critical roles in mediating the binding of SENP1 to the SUMO1-peptide. Our findings demonstrate that Model 1 not only features a greater number of strong electrostatic interactions between SENP1 and the peptide (K739) but also involves a higher number of amino acid residues that significantly contribute to the binding of SENP1 to the SUMO1-peptide. These results suggest that the amino acid environment surrounding the K739 site of nNOS is more conducive to SENP1 binding to the SUMO1-peptide. RMSF calculations further revealed the increased flexibility of certain SENP1 amino acid residues within the SENP1 and SUMO1 K739 peptide-binding model. Correlation coefficient analysis demonstrated a positive correlation between SENP1 and SUMO1 K739 peptide, whereas no such correlation was evident in the combined SENP1 and SUMO1 K725 peptide model. Collectively, the results indicated a preference of SENP1 for binding to the K739 site during nNOS deSUMOylation.

Given that SENP1 catalyzes the deSUMOylation of nNOS, our study demonstrates that among the two SUMOylation sites on nNOS, K739 and K725, SENP1 preferentially binds to the K739 site, where it exerts its catalytic activity. This finding suggested a potential strategy for designing proteins that target nNOS CaMBD to selectively interfere with SUMOylation over-activation, potentially offering therapeutic avenues for diseases linked to excessive nNOS activity. However, several significant challenges remain to be resolved. Based on all the existing findings, we hypothesized that the K739 site in nNOS CaMBD is more susceptible to SUMOylation than the K725 site. Notably, the K739 site serves not only as the primary site for nNOS SUMOylation but also as one of the key binding sites for CaM within the CaMBD domain, indicating a structural competition between these two processes. Experimental studies using artificially designed CaMBD-based peptides revealed divergent outcomes at the cellular and *in vivo* levels, suggesting that CaM may exert a mild stimulatory effect on nNOS activity, whereas SUMOylation has a more pronounced promotional effect. However, the current findings are limited to the activating roles of CaM and SUMOylation on nNOS, and the regulation of nNOS activity likely involves more intricate molecular mechanisms. Targeting a single regulatory pathway may not yield optimal clinical outcomes. Furthermore, despite the high structural similarity among human, mouse, and rat nNOS (with the latter being the primary sequence used in this study), the possibility of more complex regulatory mechanisms in human nNOS cannot be ruled out. This necessitates careful consideration of potential differences in the design of regulatory agents. The hypothesis regarding K739 susceptibility would require further support from additional simulations and calculation data. Moreover, since both CaM allosteric activation and SUMOylation occur within nNOS CaMBD, competitive interactions between these processes remain poorly understood. Further research is required to effectively modulate these pathways.

## Methods

### Model building

The initial models were developed using the X-ray crystallographic structure of SENP1 complexed with SUMO1 and RanGAP (PDB code: 2IY0). The RanGAP segment was subsequently excised from the structure. The CaMBD portion was homology-modelled based on the eNOS/CaMBD complex structure (PDB code: 2LL7). Molecular docking studies were conducted using the Z-DOCK program (version 3.0.2), as described previously (Pierce et al., 2011). The conformations in which K725 or K739 had the potential to form a covalent bond with SUMO1 were selected for further molecular dynamics simulations. The minimum potential energy conformations of the system were extracted for subsequent cluster analyses, with particular attention to those exhibiting the highest Z-DOCK scores. Representative conformations were verified for consistency with the structures generated using Coot (Emsley et al., 2010).

### Molecular dynamics simulations

All molecular dynamics simulations were executed using the AMBER 24 software with Amber19SB all-atom force field parameters (Case, 2024; Case et al., 2023; Tian et al., 2020). To achieve an ionic strength of 100 mmol/L, appropriate quantities of K^+^ and Cl^−^ ions were introduced, and each system was solvated using the TIP3P water model within a water box, maintaining a minimum solute-wall distance of 12 Å. The simulation procedures were applied uniformly across all systems. Initially, the potential energy of each system was minimized to eliminate unfavorable interactions. This process involved four rounds of minimization for a total of 2,500 steps. The first two rounds employed the steepest descent and conjugate gradient methods with restraints applied to the entire system, except for the water molecules and ions. In subsequent rounds, the system was allowed to relax without restraint. Non-bonded interactions were truncated at 12 Å, and the SHAKE algorithm was used to constrain hydrogen-containing bonds. Following minimization, the system was heated from 0 to 300 K over 200 ps under constant pressure (1 atm), with protein atom positions restrained using a force constant of 10 kcal/(mol × Å^2^). A time step of 2 fs was utilized during the heating phase. Conventional molecular dynamics simulations were conducted for 500 ns without restraints. The same simulation protocols were applied to the other systems. Free energy calculations were performed using the MMPBSA. py script in AmberTools employing the MM-GBSA implicit solvent model while maintaining a fixed ionic strength of 100 mmol/L. Additional analyses, including polar contacts, hydrogen bonds, and correlation analyses, were conducted using the CPPTRAJ module, following established methods reported in previous studies (Cheng and Wang, 2022; Roe and Cheatham, 2013).

### Establishment of the MCAO model

Mice were anesthetized via intraperitoneal injection of sodium pentobarbital (30 mg/kg, 0.15 mL/10 g body weight). Following confirmation of anesthesia, mice were positioned supine on a surgical board, and the neck skin was disinfected with 75% ethanol. A midline incision of approximately 1 cm was made along the neck, followed by blunt dissection of subcutaneous tissues, muscles, and fascia to expose the right common carotid artery (CCA). The CCA was carefully isolated to its bifurcation, revealing the internal carotid artery (ICA) and external carotid artery (ECA), with a 4–0 sterile surgical suture pre-placed for subsequent use. The ICA was temporarily occluded using a microarterial clip, and the proximal ends of the CCA and ECA were ligated with 6–0 sterile silk sutures. A small incision (approximately 0.5 mm) was made near the CCA bifurcation, through which a heparin-coated nylon suture (diameter 0.16 mm, tip diameter 0.20 ± 0.01 mm, length approximately 20 mm) was slowly inserted, advanced through the CCA and ICA to the origin of the middle cerebral artery (MCA), thereby occluding blood flow to the right MCA. The insertion depth of the suture, measured from the ICA-ECA bifurcation, was 10 ± 0.5 mm. The ICA was ligated with a 6–0 silk suture to secure the suture and prevent intraoperative bleeding. After maintaining occlusion for 30 min, the suture was slowly withdrawn to achieve reperfusion. The CCA incision was sutured with a 6–0 silk suture, the microarterial clip was removed to restore ICA blood flow, and mouse behavior and vital signs were monitored for 24 h post-surgery.

### Morris water maze experiment under peptide influence

Male C57BL/6 mice, aged 4–6 weeks (body weight 22–25 g), were randomly assigned into seven groups (n = 6 per group) based on body weight: sham, control, and experimental groups 1–5. Purified peptides (purchased from Sangon Biotech, see supplementary) for the control and experimental groups 1–5 were dissolved in saline to a final concentration of 12.5 μg/μL (1 mg peptide in 80 μL saline). Using a stereotaxic injector, 1 μL of peptide solution (total 25 μg peptide per mouse) was administered bilaterally into the hippocampal CA1 region and cortical M1 region of each mouse. Two hours post-injection, all groups except the sham group underwent the middle cerebral artery occlusion (MCAO) model procedure. On the third day post-MCAO, the Morris water maze test was conducted to evaluate the effects of peptides on spatial learning and memory. The training phase spanned 5 days, with four trials per day, during which mice were released from random starting points in different quadrants, and the latency to locate the platform (up to 60 s) was recorded. If a mouse failed to locate the platform, it was guided to the platform and allowed to remain for 15 s. On the sixth day, the platform was removed, and the time spent in the target quadrant and the number of platform crossings were recorded. Differences between groups were analyzed using one-way analysis of variance (ANOVA).

### TTC staining experiment in the MCAO model with peptide injection

Male C57BL/6 mice, aged 4–6 weeks (body weight 25 ± 2 g), were randomly divided into four groups (n = 6 per group): sham, control (ischemia/reperfusion, I/R), and model + experimental groups 1 and 2. Experimental peptides 1 and 2 were dissolved in saline to a final concentration of 12.5 μg/μL (1 mg peptide in 80 μL saline). Using a stereotaxic injector, 1 μL of peptide solution (total 25 μg peptide per mouse) was administered into the hippocampal CA1 region and cortical M1 region of each mouse. Two hours after the injection, all groups except the sham group underwent the MCAO model procedure. 24 h after MCAO, mouse brains were extracted and coronally sectioned into 6–8 uniform slices. The slices were incubated in a 2% 2,3,5-triphenyltetrazolium chloride (TTC) solution (dissolved in PBS, pH 7.4) at 37 °C in the dark for 15–30 min, with gentle shaking to ensure uniform staining. Healthy tissue stained red, while infarcted areas appeared white. Slices were fixed in 4% paraformaldehyde, photographed, and the infarct area of each slice was calculated. Total infarct volume (mm^3^) was determined by summing the infarct areas and multiplying by slice thickness. Relative infarct volume was expressed as the percentage of infarct volume relative to the whole brain or hemisphere volume.

All mouse experiments described herein were reviewed and approved by the Laboratory Animal Ethics Committee of Xuzhou Medical University (Process number: 202112A385; see attachments).

## Data Availability

The original contributions presented in the study are included in the article/Supplementary Material, further inquiries can be directed to the corresponding author.
