# Supplementary material for: K739 is preferentially targeted over K725 in the deSUMOylation process of neuronal nitric oxide synthase
Source: Front Chem. 2025 Sep 4;13:1672437. doi: 10.3389/fchem.2025.1672437 (PMC12445055; doi:10.3389/fchem.2025.1672437)
Supplement: Supplementary file 1 [file DataSheet1.pdf]

生工® Sangon Biotech

Sample Information

Name	: P30536-1（control）
Sequence	: YGRKKRRQRRREAKLKAFFAKSLKV
Modification	: N/A
Lot.No	: P30536-1（control）-23092501
Pump A	: 0.1%trifluoroacetic in 100%water
Pump B	: 0.1%trifluoroacetic in 100%acetonrtrile
Total Flow	: 1.0ml/min
Wavelength	: 214nm
Analytical column type	: SHIMADZU shim-pack GIST(4.6*250MM*5UM)
Dissolution method	: 0.1mg sample dissolved to 0.5mL by 10%HCOOH 20%ACN and 70%H2O
Acquisition Time	: 2023/10/26 12:20:58
Inj.Volume	: 30ul

Time	Module	Action	Value
0.01	Pumps	B.Conc	25
20.00	Pumps	B.Conc	45

Chromatogram

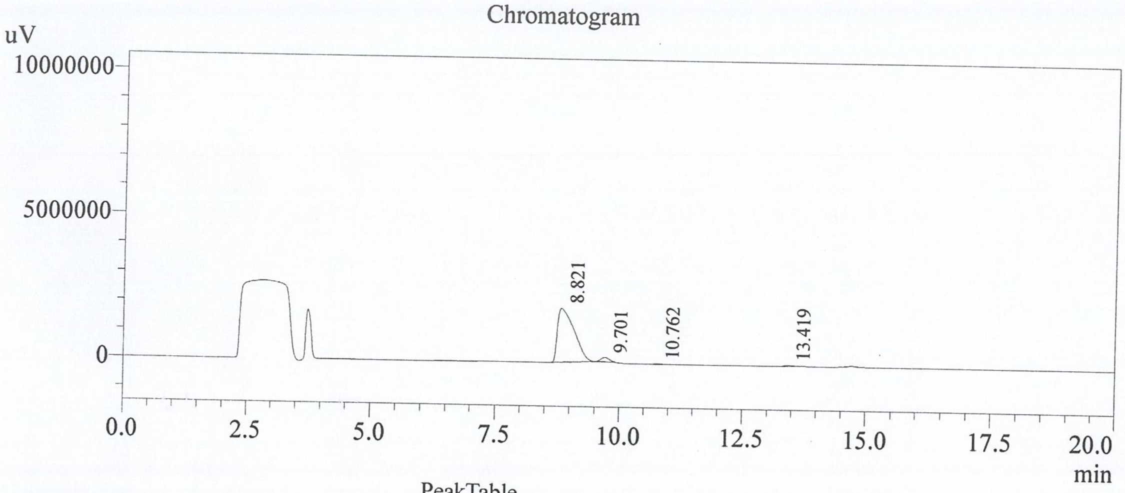

PeakTable

检测器A 214nm

Peak#	Ret. Time	Area	Height	Area %	Height %
1	8.821	43338027	1864750	95.199	90.765
2	9.701	1802537	151797	3.960	7.389
3	10.762	106517	11615	0.234	0.565
4	13.419	276305	26327	0.607	1.281
总计		45523385	2054489	100.000	100.000

生工生物工程（上海）股份有限公司

地址: 上海市松江区香闵路698号
电话/Tel: 400-821-0268
邮箱/Email: sales@sangon.com

Add: 698 Xiang Min Road SongJiang Shanghai China
传真/Fax: 86-21-37772170
网址/Web: www.sangon.com

生工® Sangon Biotech

MS Spectrum

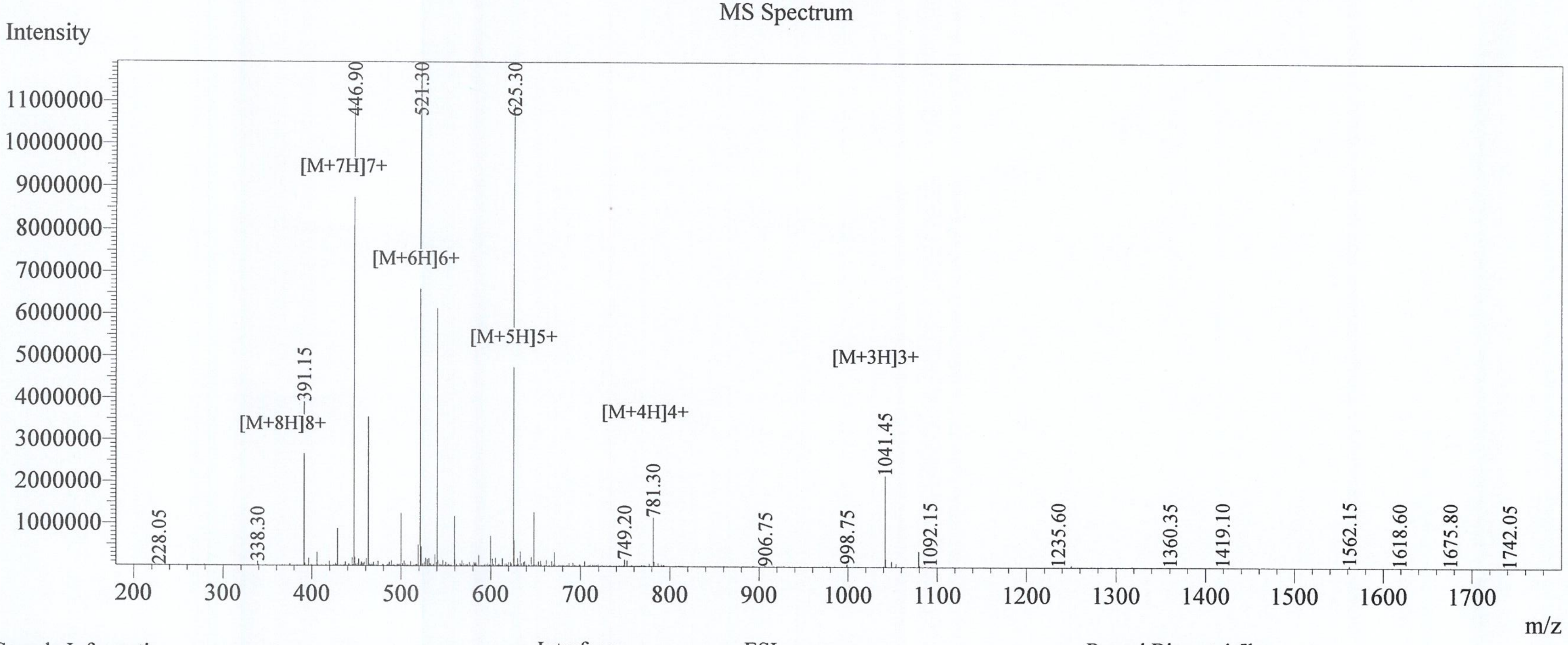

Sample Information
Dissolution method :0.1mg sample dissolved to 0.5mL by 50%ACN and 50%H2O
Date Acquired :2023/10/26 13:09:23
Injection Volume :1ul

Interface :ESI
Nebulizing Gas Flow :1.50L/min
CDL Temp :250℃
CDL Volt :0v
Block Temp :200

Prerod Bias :+4.5kv
Detector :-0.2kv
T.Flow :0.2ml/min
B.conc :50%H2O/50%MeOH

Name :P30536-1（control）
Sequence :YGRKKRRQRRREAKLKAFFAKSLKV
Modification :N/A
Lot No. :P30536-1（control）-23092501
Theoretical :3121.745
bserved :3121.80

生工生物工程（上海）股份有限公司

地址：上海市松江区香闵路698号
电话/Tel: 400-821-0268
邮箱/Email: Sales@sangon.com

Add: 698 Xiang Min Road Songjiang Shanghai China
传真/Fax: 86-21-37772170
网址/Web: wwww.sangon.com

生工® Sangon Biotech

多肽合成报告单

CERTIFICATE OF ANALYSIS

Product Name	P30536-1（control）
Catalog No.	N/A
Lot No.	P30536-1（control）-23092501
Sequence	YGRKKRRQRRREAKLKAFFAKSLKV
Length	25AA
Modification	N/A
Molecular Weight (MW)	3121.75
Storage	-20°C

Test Items	Specifications	Results
MW by MS	3121.8	Conforms
Purity by HPLC	> 95%脱盐	95.199%脱盐
Peptide Content	N/A	N/A
Acetic acid content	N/A	N/A
Appearance	White to off-white lyophilized powder	Conforms
Quantity	5mg	5.0mg

Certified by: Melinda   Date 10/26/2023

Quality Assurance Department

生工® Sangon Biotech

Sample Information

Name : P30536-2（1）
Sequence : YGRKKRRQRRRFKKLAEAVKFSAKL
Modification : N/A
Lot.No : P30536-2（1）-23092501

Pump A : 0.1%trifluoroacetic in 100%water
Pump B : 0.1%trifluoroacetic in 100%acetonrtrile
Total Flow : 1.0ml/min
Wavelength : 214nm
Analytical column type : SHIMADZU shim-pack GIST(4.6*250MM*5UM)
Dissolution method : 0.1mg sample dissolved to 0.5mL by 20%ACN and 80%H2O
Acquisition Time : 2023/10/26 11:35:44
Inj.Volume : 30ul

Time	Module	Action	Value
0.01	Pumps	B.Conc	22
20.00	Pumps	B.Conc	42

Chromatogram

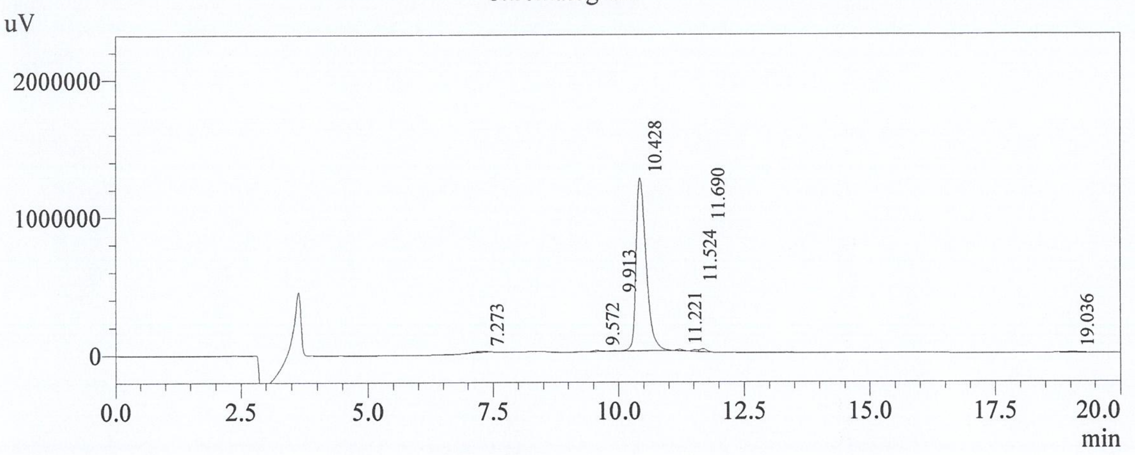

PeakTable

检测器A 214nm

Peak#	Ret. Time	Area	Height	Area %	Height %
1	7.273	138887	8306	0.759	0.629
2	9.572	88301	8105	0.483	0.614
3	9.913	26970	3931	0.147	0.298
4	10.428	17576187	1251277	96.115	94.831
5	11.221	76249	9980	0.417	0.756
6	11.524	86658	11039	0.474	0.837
7	11.690	190470	21898	1.042	1.660
8	19.036	102952	4939	0.563	0.374
总计		18286674	1319476	100.000	100.000

生工生物工程（上海）股份有限公司

地址: 上海市松江区香闵路698号
电话/Tel: 400-821-0268
邮箱/Email: sales@sangon.com

Add: 698 Xiang Min Road SongJiang Shanghai China
传真/Fax: 86-21-37772170
网址/Web: www.sangon.com

生工® Sangon Biotech

MS Spectrum

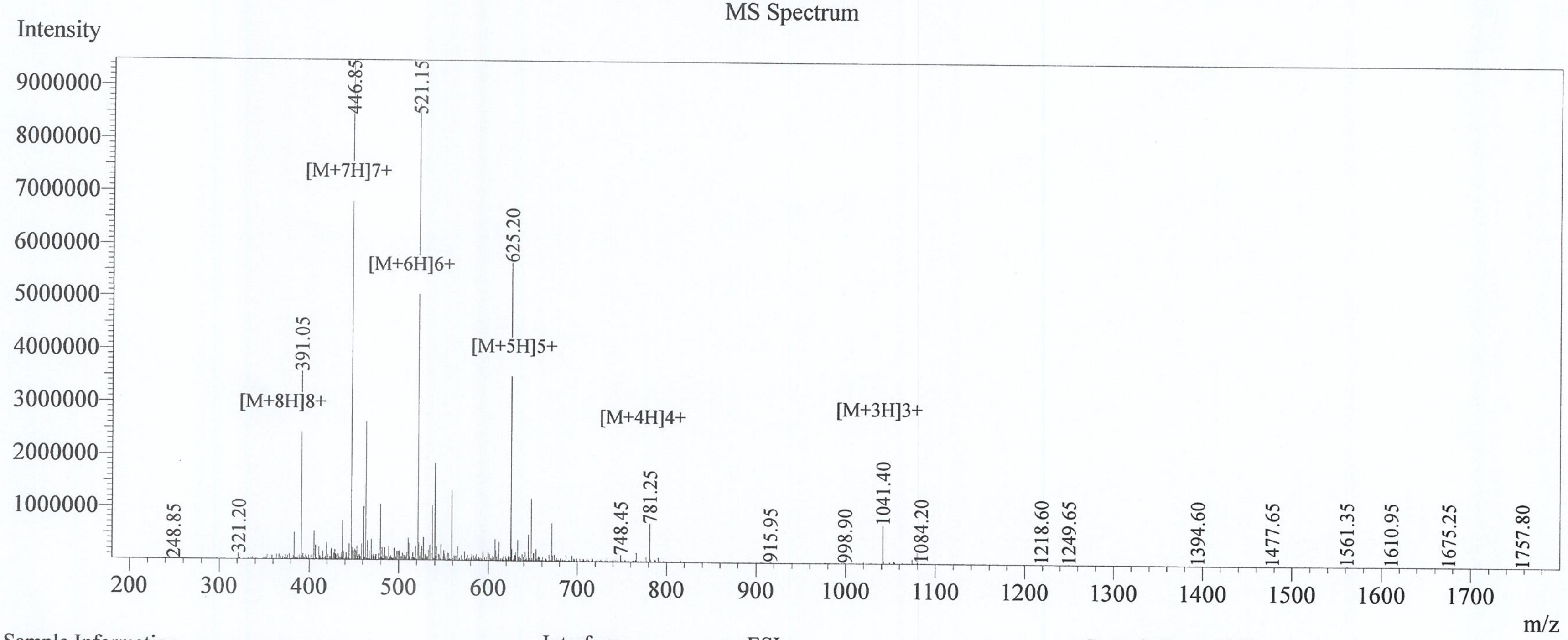

Sample Information
Dissolution method :0.1mg sample dissolved to 0.5mL by 50%ACN and 50%H2O
Date Acquired :2023/10/26 09:44:39
Injection Volume :1ul
Name :P30536-2（1）
Sequence :YGRKKRRQRRRFKKLAEAVKFSAKL
Modification :N/A
Lot No. :P30536-2（1）-23092501
Theoretical :3121.745
bserved :3120.95

Interface :ESI
Nebulizing Gas Flow :1.50L/min
CDL Temp :250°C
CDL Volt :0v
Block Temp :200

Prerod Bias :+4.5kv
Detector :-0.2kv
T.Flow :0.2ml/min
B.conc :50%H2O/50%MeOH

生工生物工程（上海）股份有限公司

地址：上海市松江区香闵路698号
电话/Tel: 400-821-0268
邮箱/Email: Sales@sangon.com

Add: 698 Xiang Min Road Songjiang Shanghai China
传真/Fax: 86-21-37772170
网址/Web: wwww.sangon.com

生工® Sangon Biotech 多肽合成报告单

CERTIFICATE OF ANALYSIS

**Product Name**	P30536-2（1）
**Catalog No.**	N/A
**Lot No.**	P30536-2（1）-23092501
**Sequence**	YGRKKRRQRRRFKKLAEAVKFSAKL
**Length**	25AA
**Modification**	N/A
**Molecular Weight (MW)**	3121.75
**Storage**	-20°C

**Test Items**	**Specifications**	**Results**
**MW by MS**	3121.0	Conforms
**Purity by HPLC**	＞95%脱盐	96.115%脱盐
**Peptide Content**	N/A	N/A
**Acetic acid content**	N/A	N/A
**Appearance**	White to off-white lyophilized powder	Conforms
**Quantity**	5mg	5.0mg

Certified by: Melinda　　Date 10/26/2023

Quality Assurance Department

生工® Sangon Biotech

Sample Information

Name	:	P30536-3（2）
Sequence	:	YGRKKRRQRRRFKKFAEAVKFSAKL
Modification	:	N/A
Lot.No	:	P30536-3（2）-23092501
Pump A	:	0.1%trifluoroacetic in 100%water
Pump B	:	0.1%trifluoroacetic in 100%acetonrtrile
Total Flow	:	1.0ml/min
Wavelength	:	214nm
Analytical column type	:	SHIMADZU shim-pack GIST(4.6*250MM*5UM)
Dissolution method	:	0.1mg sample dissolved to 0.5mL by 10%ACN and 90%H2O
Acquisition Time	:	2023/10/25 10:40:59
Inj.Volume	:	30ul

Time	Module	Action	Value
0.01	Pumps	B.Conc	22
20.00	Pumps	B.Conc	42

Chromatogram

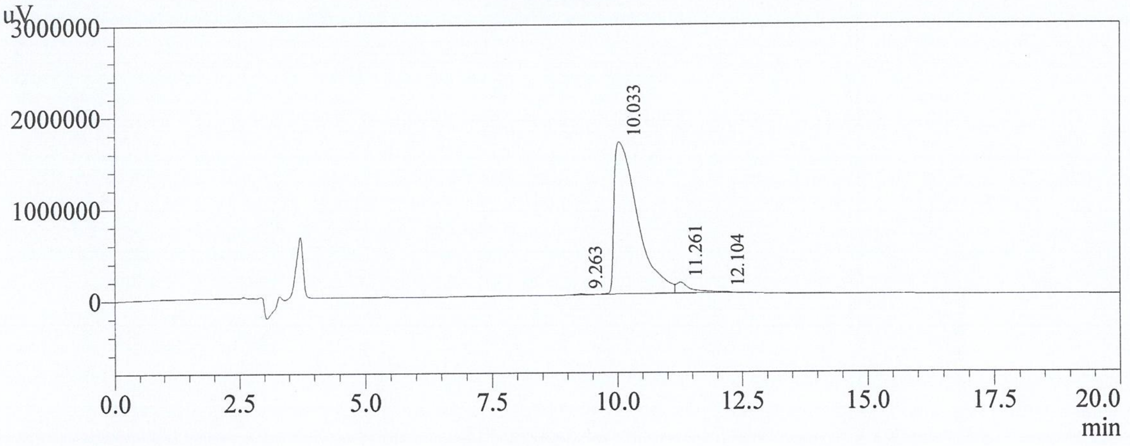

1 Det.A Ch1 / 214nm

PeakTable

Detector A Ch1 214nm

Peak#	Ret. Time	Area	Height	Area %	Height %
1	9.263	112682	10779	0.199	0.600
2	10.033	53900868	1659707	95.344	92.312
3	11.261	2414359	119045	4.271	6.621
4	12.104	105354	8402	0.186	0.467
Total		56533263	1797933	100.000	100.000

生工生物工程（上海）股份有限公司

地址: 上海市松江区香闵路698号
电话/Tel: 400-821-0268
邮箱/Email: sales@sangon.com

Add: 698 Xiang Min Road SongJiang Shanghai China
传真/Fax: 86-21-37772170
网址/Web: www.sangon.com

生工® Sangon Biotech

MS Spectrum

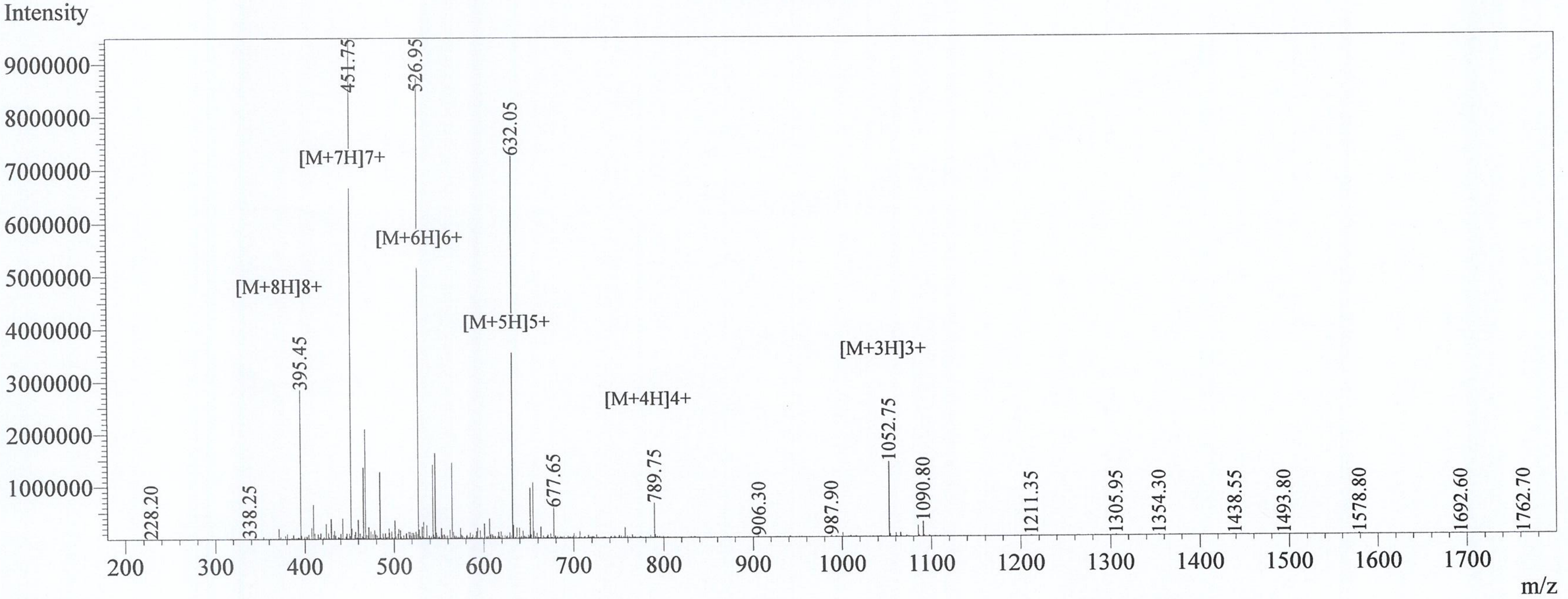

Sample Information
Dissolution method :0.1mg sample dissolved to 0.5mL by 50%ACN and 50%H2O
Date Acquired :2023/10/25 12:35:40
Injection Volume :1ul
Name :P30536-3（2）
Sequence :YGRKKRRQRRRFKKFAEAVKFSAKL
Modification :N/A
Lot No. :P30536-3（2）-23092501
Theoretical :3155.762
bserved :3155.25

Interface :ESI
Nebulizing Gas Flow :1.50L/min
CDL Temp :250°C
CDL Volt :0v
Block Temp :200

Prerod Bias :+4.5kv
Detector :-0.2kv
T.Flow :0.2ml/min
B.conc :50%H2O/50%MeOH

生工生物工程（上海）股份有限公司

地址：上海市松江区香闵路698号
电话/Tel: 400-821-0268
邮箱/Email: Sales@sangon.com

Add: 698 Xiang Min Road Songjiang Shanghai China
传真/Fax: 86-21-37772170
网址/Web: www.sangon.com

CERTIFICATE OF ANALYSIS

**Product Name**	P30536-3（2）
**Catalog No.**	N/A
**Lot No.**	P30536-3（2）-23092501
**Sequence**	YGRKKRRQRRRFKKFAEAVKFSAKL
**Length**	25AA
**Modification**	N/A
**Molecular Weight (MW)**	3155.76
**Storage**	-20°C

**Test Items**	**Specifications**	**Results**
**MW by MS**	3155.3	Conforms
**Purity by HPLC**	> 95%脱盐	95.344%脱盐
**Peptide Content**	N/A	N/A
**Acetic acid content**	N/A	N/A
**Appearance**	White to off-white lyophilized powder	Conforms
**Quantity**	5mg	5.0mg

Certified by: Melinda Date 10/25/2023

Quality Assurance Department

生工® Sangon Biotech

Sample Information

Name : P30536-4（3）
Sequence : YGRKKRRQRRRYKKLAEAVKYSAKL
Modification : N/A
Lot.No : P30536-4（3）-23092501

Pump A : 0.1%trifluoroacetic in 100%water
Pump B : 0.1%trifluoroacetic in 100%acetonrtrile
Total Flow : 1.0ml/min
Wavelength : 214nm
Analytical column type : SHIMADZU shim-pack GIST(4.6*250MM*5UM)
Dissolution method : 0.1mg sample dissolved to 0.5mL by 10%ACN and 90%H2O
Acquisition Time : 2023/10/25 12:26:36
Inj.Volume : 30ul

Time	Module	Action	Value
0.01	Pumps	B.Conc	17
20.00	Pumps	B.Conc	37

Chromatogram

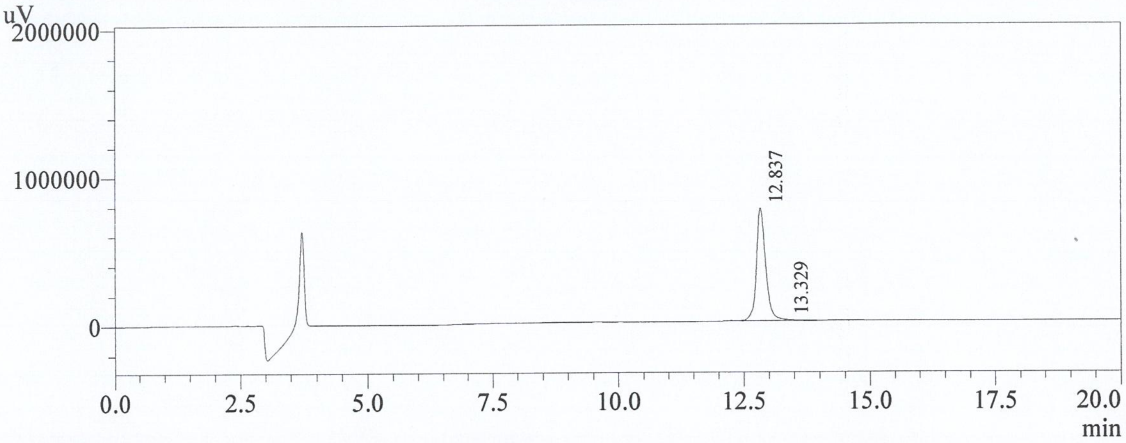

PeakTable

检测器A 214nm

Peak#	Ret. Time	Area	Height	Area %	Height %
1	12.837	9617340	755951	98.785	98.909
2	13.329	118249	8336	1.215	1.091
总计		9735589	764287	100.000	100.000

生工生物工程（上海）股份有限公司

地址: 上海市松江区香闵路698号
电话/Tel: 400-821-0268
邮箱/Email: sales@sangon.com

Add: 698 Xiang Min Road SongJiang Shanghai China
传真/Fax: 86-21-37772170
网址/Web: www.sangon.com

生工® Sangon Biotech

MS Spectrum

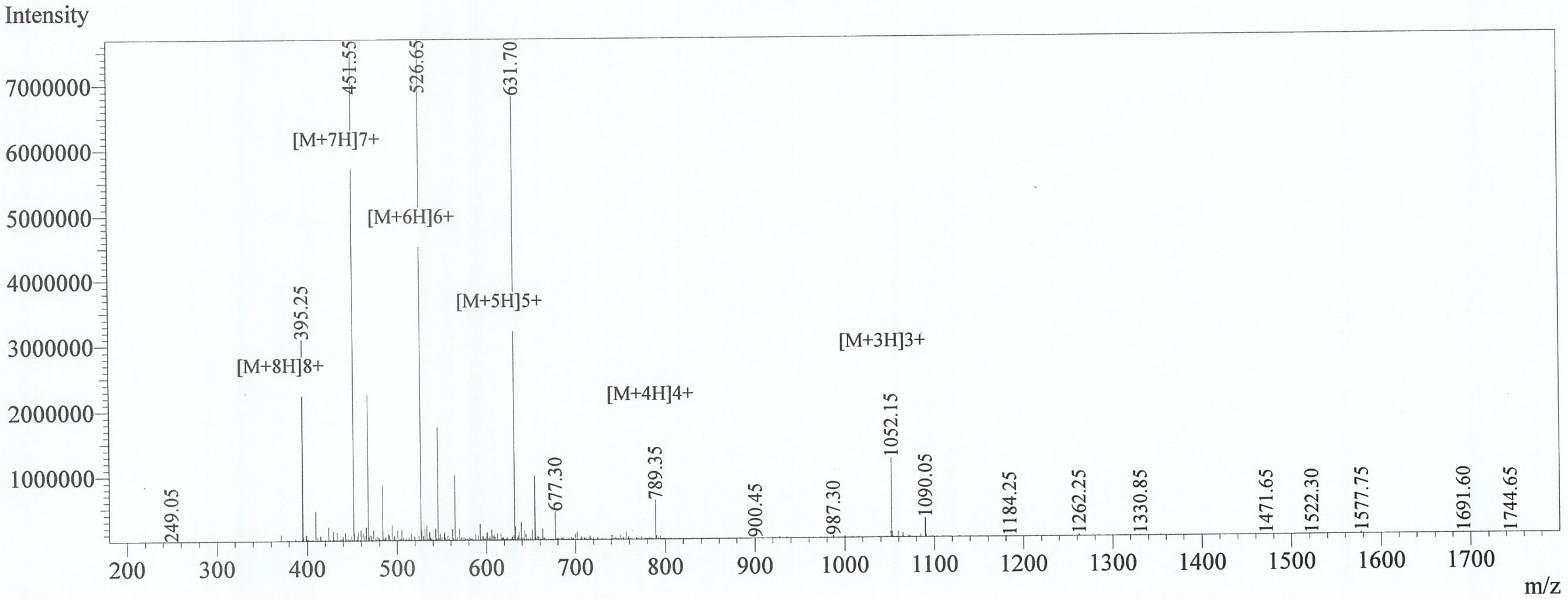

Sample Information
Dissolution method :0.1mg sample dissolved to 0.5mL by 50%ACN and 50%H2O
Date Acquired :2023/10/25 09:53:25
Injection Volume :1ul
Name :P30536-4（3）
Sequence :YGRKKRRQRRRYKKLAEAVKYSAKL
Modification :N/A
Lot No. :P30536-4（3）-23092501
Theoretical :3153.743
bserved :3153.90

Interface :ESI
Nebulizing Gas Flow :1.50L/min
CDL Temp :250°C
CDL Volt :0v
Block Temp :200

Prerod Bias :+4.5kv
Detector :-0.2kv
T.Flow :0.2ml/min
B.conc :50%H2O/50%MeOH

生工生物工程（上海）股份有限公司

地址：上海市松江区香闵路698号
电话/Tel: 400-821-0268
邮箱/Email: Sales@sangon.com

Add: 698 Xiang Min Road Songjiang Shanghai China
传真/Fax: 86-21-37772170
网址/Web: www.sangon.com

生工® Sangon Biotech　　多肽合成报告单

CERTIFICATE OF ANALYSIS

**Product Name**	P30536-4（3）
**Catalog No.**	N/A
**Lot No.**	P30536-4（3）-23092501
**Sequence**	YGRKKRRQRRRYKKLAEAVKYSAKL
**Length**	25AA
**Modification**	N/A
**Molecular Weight (MW)**	3153.74
**Storage**	-20°C

**Test Items**	**Specifications**	**Results**
**MW by MS**	3153.9	Conforms
**Purity by HPLC**	＞95%脱盐	98.785%脱盐
**Peptide Content**	N/A	N/A
**Acetic acid content**	N/A	N/A
**Appearance**	White to off-white lyophilized powder	Conforms
**Quantity**	5mg	5.0mg

Certified by: Melinda　　Date 10/25/2023

Quality Assurance Department

生工® Sangon Biotech

Sample Information

Name : P30536-5（4）
Sequence : YGRKKRRQRRRLKKLAEALKLSAKL
Modification : N/A
Lot.No : P30536-5（4）-23092501
Pump A : 0.1%trifluoroacetic in 100%water
Pump B : 0.1%trifluoroacetic in 100%acetonrtrile
Total Flow : 1.0ml/min
Wavelength : 214nm
Analytical column type : SHIMADZU shim-pack GIST(4.6*250MM*5UM)
Dissolution method : 0.1mg sample dissolved to 0.5mL by 10%ACN and 90%H2O
Acquisition Time : 2023/10/25 09:59:38
Inj.Volume : 30ul

Time	Module	Action	Value
0.01	Pumps	B.Conc	23
20.00	Pumps	B.Conc	43

Chromatogram

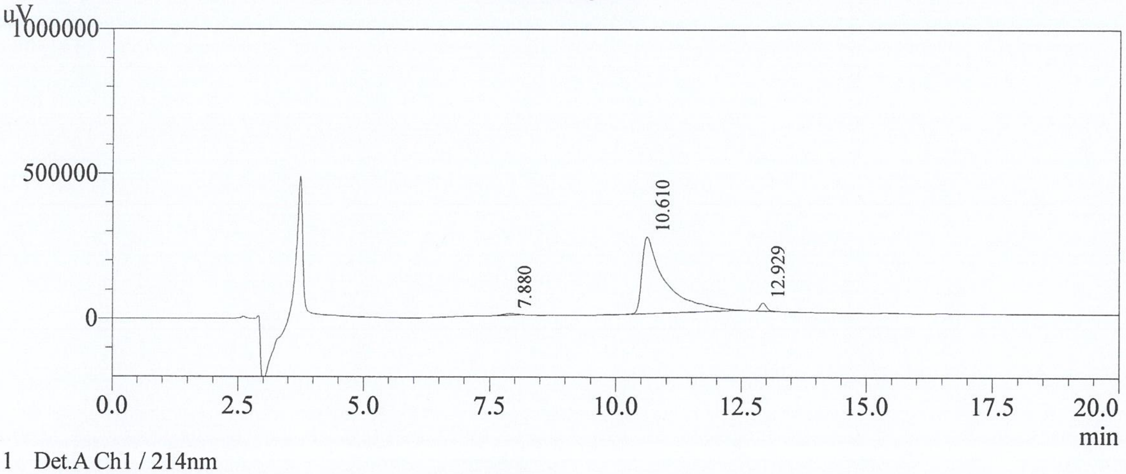

1 Det.A Ch1 / 214nm

PeakTable

Detector A Ch1 214nm

Peak#	Ret. Time	Area	Height	Area %	Height %
1	7.880	90055	5415	1.002	1.826
2	10.610	8632995	262940	96.100	88.652
3	12.929	260317	28244	2.898	9.523
Total		8983368	296599	100.000	100.000

生工生物工程（上海）股份有限公司

地址: 上海市松江区香闵路698号
电话/Tel: 400-821-0268
邮箱/Email: sales@sangon.com

Add: 698 Xiang Min Road SongJiang Shanghai China
传真/Fax: 86-21-37772170
网址/Web: www.sangon.com

生工® Sangon Biotech

MS Spectrum

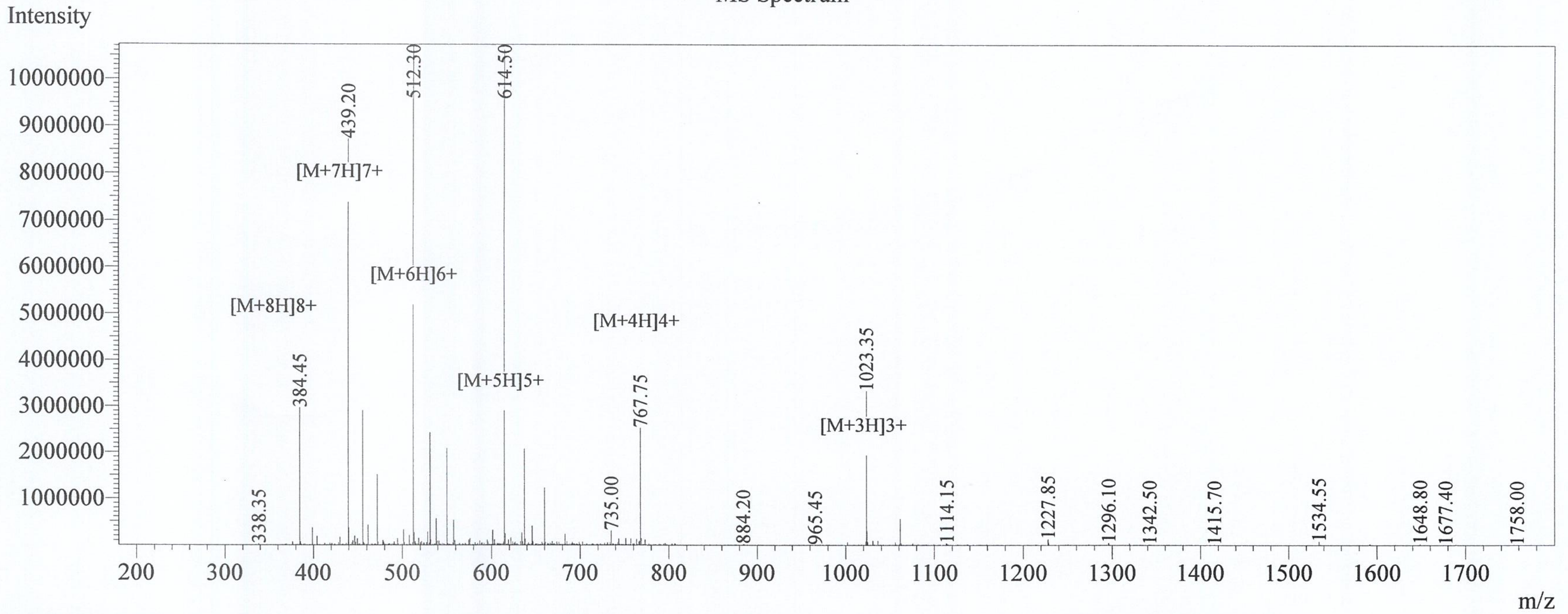

Sample Information
Dissolution method :0.1mg sample dissolved to 0.5mL by 50%ACN and 50%H2O
Date Acquired :2023/10/25 11:12:01
Injection Volume :1ul
Name :P30536-5（4）
Sequence :YGRKKRRQRRRLKKLAEALKLSAKL
Modification :N/A
Lot No. :P30536-5（4）-23092501
Theoretical :3067.738
bserved :3067.80

Interface :ESI
Nebulizing Gas Flow :1.50L/min
CDL Temp :250℃
CDL Volt :0v
Block Temp :200

Prerod Bias :+4.5kv
Detector :-0.2kv
T.Flow :0.2ml/min
B.conc :50%H2O/50%MeOH

生工生物工程（上海）股份有限公司

地址：上海市松江区香闵路698号
电话/Tel: 400-821-0268
邮箱/Email: Sales@sangon.com

Add: 698 Xiang Min Road Songjiang Shanghai China
传真/Fax: 86-21-37772170
网址/Web: www.sangon.com

生工® Sangon Biotech 多肽合成报告单

CERTIFICATE OF ANALYSIS

**Product Name**	P30536-5（4）
**Catalog No.**	N/A
**Lot No.**	P30536-5（4）-23092501
**Sequence**	YGRKKRRQRRRLKKLAEALKLSAKL
**Length**	25AA
**Modification**	N/A
**Molecular Weight (MW)**	3067.74
**Storage**	-20°C

**Test Items**	**Specifications**	**Results**
**MW by MS**	3067.8	Conforms
**Purity by HPLC**	> 95%脱盐	96.100%脱盐
**Peptide Content**	N/A	N/A
**Acetic acid content**	N/A	N/A
**Appearance**	White to off-white lyophilized powder	Conforms
**Quantity**	5mg	5.0mg

Certified by: Melinda   Date 10/25/2023

Quality Assurance Department

生工® Sangon Biotech

Sample Information

Name : P30536-6（5）
Sequence : YGRKKRRQRRRLVKAVFFASVLM
Modification : N/A
Lot.No : P30536-6（5）-23092501

Pump A : 0.1%trifluoroacetic in 100%water
Pump B : 0.1%trifluoroacetic in 100%acetonrtrile
Total Flow : 1.0ml/min
Wavelength : 214nm
Analytical column type : SHIMADZU Inertsil ODS-SP(4.6*250MM*5UM)
Dissolution method : 0.1mg sample dissolved to 0.5mL by 10%ACN and 90%H2O
Acquisition Time : 2023/10/26 09:56:21
Inj.Volume : 10ul

Time	Module	Action	Value
0.01	Pumps	B.Conc	28
20.00	Pumps	B.Conc	48

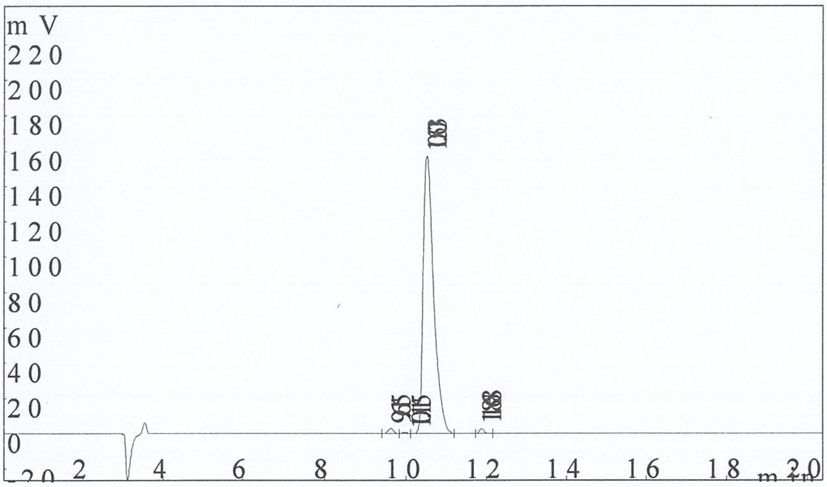

PeakTable

Detector A Ch 214nm

Peak	Ret.Time	Area	Height	Area%	Height%
1	9.615	29101	3034	1.042	1.862
2	10.115	7878	0	0.282	0.000
3	10.503	2738359	157014	98.013	96.342
4	11.868	18545	2928	0.663	1.796
Total		2793883	162976	100.000	100.000

生工生物工程（上海）股份有限公司

地址: 上海市松江区香闵路698号
电话/Tel: 400-821-0268
邮箱/Email: sales@sangon.com
Add: 698 Xiang Min Road SongJiang Shanghai China
传真/Fax: 86-21-37772170
网址/Web: www.sangon.com

生工® Sangon Biotech

MS Spectrum

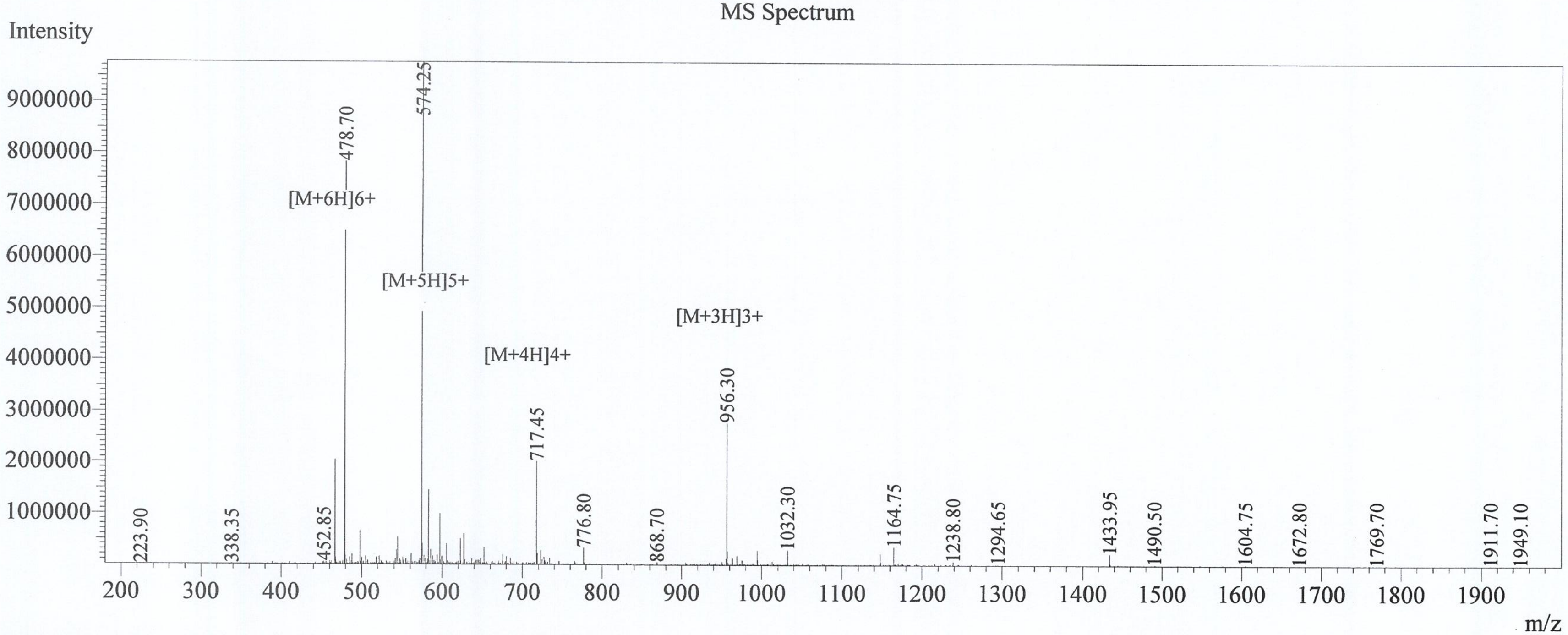

Sample Information
Dissolution method :0.1mg sample dissolved to 0.5mL by 50%ACN and 50%H2O
Date Acquired :2023/10/26 10:58:48
Injection Volume :1ul
Name :P30536-6（5）
Sequence :YGRKKRRQRRRLVKAVFFASVLM
Modification :N/A
Lot No. :P30536-6（5）-23092501
Theoretical :2866.505
bserved :2866.25

Interface :ESI
Nebulizing Gas Flow :1.50L/min
CDL Temp :250°C
CDL Volt :0v
Block Temp :200

Prerod Bias :+4.5kv
Detector :-0.2kv
T.Flow :0.2ml/min
B.conc :50%H2O/50%MeOH

生工生物工程（上海）股份有限公司

地址：上海市松江区香闵路698号
电话/Tel: 400-821-0268
邮箱/Email: Sales@sangon.com

Add: 698 Xiang Min Road Songjiang Shanghai China
传真/Fax: 86-21-37772170
网址/Web: www.sangon.com

生工® Sangon Biotech 多肽合成报告单

CERTIFICATE OF ANALYSIS

**Product Name**	P30536-6（5）
**Catalog No.**	N/A
**Lot No.**	P30536-6（5）-23092501
**Sequence**	YGRKKRRQRRRLVKAVFFASVLM
**Length**	23AA
**Modification**	N/A
**Molecular Weight (MW)**	2866.51
**Storage**	-20°C

**Test Items**	**Specifications**	**Results**
**MW by MS**	2866.3	Conforms
**Purity by HPLC**	> 95%脱盐	98.013%脱盐
**Peptide Content**	N/A	N/A
**Acetic acid content**	N/A	N/A
**Appearance**	White to off-white lyophilized powder	Conforms
**Quantity**	5mg	5.0mg

Certified by: Melinda   Date 10/26/2023

Quality Assurance Department

生工® Sangon Biotech

Sample Information

Name	:	P30536-7（6）
Sequence	:	YGRKKRRQRRRTFKEVANAVKISAS
Modification	:	N/A
Lot.No	:	P30536-7（6）-23092501
Pump A	:	0.1%trifluoroacetic in 100%water
Pump B	:	0.1%trifluoroacetic in 100%acetonrtrile
Total Flow	:	1.0ml/min
Wavelength	:	214nm
Analytical column type	:	SHIMADZU Inertsil ODS-SP(4.6*250MM*5UM)
Dissolution method	:	0.1mg sample dissolved to 0.5mL by 10%ACN and 90%H2O
Acquisition Time	:	2023/10/26 09:20:58
Inj.Volume	:	10ul

Time	Module	Action	Value
0.01	Pumps	B.Conc	19
20.00	Pumps	B.Conc	39

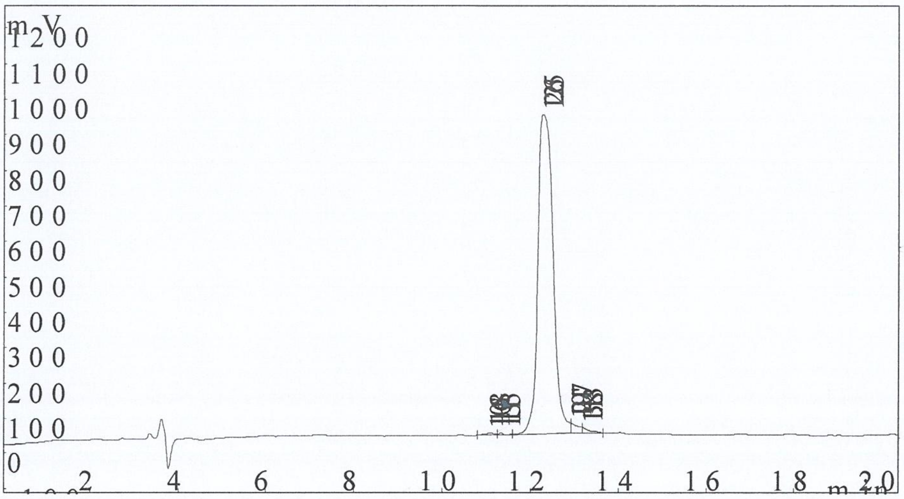

PeakTable

Detector A Ch 214nm

Peak	Ret.Time	Area	Height	Area%	Height%
1	11.068	71103	4345	0.327	0.456
2	11.315	21629	4192	0.099	0.440
3	12.265	21135469	900393	97.344	94.590
4	12.917	334547	27988	1.541	2.940
5	13.183	149642	14975	0.689	1.574
Total		21712390	951893	100.000	100.000

生工生物工程（上海）股份有限公司

地址: 上海市松江区香闵路698号
电话/Tel: 400-821-0268
邮箱/Email: sales@sangon.com
Add: 698 Xiang Min Road SongJiang Shanghai China
传真/Fax: 86-21-37772170
网址/Web: www.sangon.com

生工® Sangon Biotech

MS Spectrum

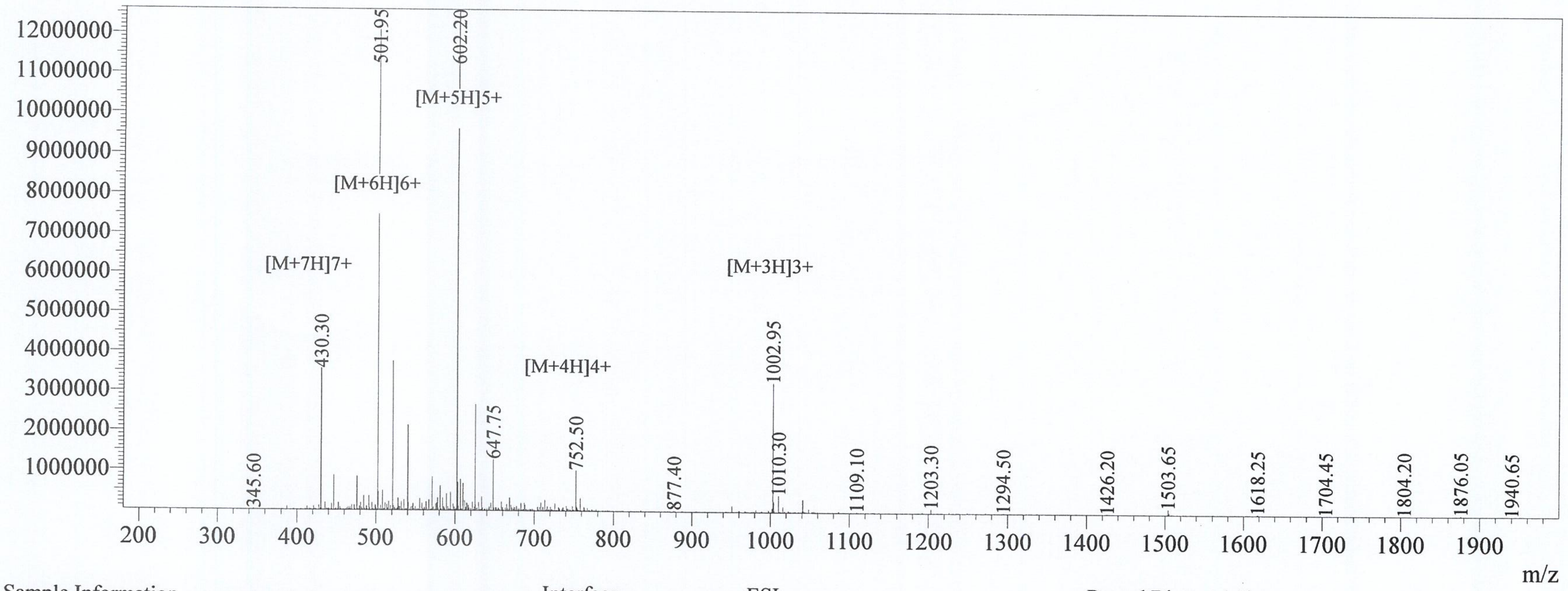

Sample Information
Dissolution method :0.1mg sample dissolved to 0.5mL by 50%ACN and 50%H2O
Date Acquired :2023/10/26 10:41:06
Injection Volume :1ul
Name :P30536-7（6）
Sequence :YGRKKRRQRRRTFKEVANAVKISAS
Modification :N/A
Lot No. :P30536-7（6）-23092501
Theoretical :3006.488
bserved :3006.00

Interface :ESI
Nebulizing Gas Flow :1.50L/min
CDL Temp :250°C
CDL Volt :0v
Block Temp :200

Prerod Bias :+4.5kv
Detector :-0.2kv
T.Flow :0.2ml/min
B.conc :50%H2O/50%MeOH

生工生物工程（上海）股份有限公司

地址：上海市松江区香闵路698号
电话/Tel: 400-821-0268
邮箱/Email: Sales@sangon.com

Add: 698 Xiang Min Road Songjiang Shanghai China
传真/Fax: 85-21-37772170
网址/Web: wwww.sangon.com

CERTIFICATE OF ANALYSIS

**Product Name**	P30536-7（6）
**Catalog No.**	N/A
**Lot No.**	P30536-7（6）-23092501
**Sequence**	YGRKKRRQRRRTFKEVANAVKISAS
**Length**	25AA
**Modification**	N/A
**Molecular Weight (MW)**	3006.49
**Storage**	-20°C

**Test Items**	**Specifications**	**Results**
**MW by MS**	3006.0	Conforms
**Purity by HPLC**	> 95%脱盐	97.344%脱盐
**Peptide Content**	N/A	N/A
**Acetic acid content**	N/A	N/A
**Appearance**	White to off-white lyophilized powder	Conforms
**Quantity**	5mg	5.0mg

Certified by: Melinda Date 10/26/2023

Quality Assurance Department

生工® Sangon Biotech

Sample Information

Name ： P30536-8（Fcontrol）
Sequence ： FITC-Acp-YGRKKRRQRRREAKLKAFFAKSLKV
Modification ： N-terminal FITC-Acp
Lot.No ： P30536-8（Fcontrol）-23092501

Pump A ： 0.1%trifluoroacetic in 100%water
Pump B ： 0.1%trifluoroacetic in 100%acetonrtrile
Total Flow ： 1.0ml/min
Wavelength ： 214nm
Analytical column type ： NanoChrom Chromcore TM120 C18(4.6*250MM*5UM)
Dissolution method ： 0.1mg sample dissolved to 0.5mL by 20%ACN and 80%H2O
Acquisition Time ： 2023/10/26 12:25:06
Inj.Volume ： 30ul

Time	Module	Action	Value
0.01	Pumps	B.Conc	28
20.00	Pumps	B.Conc	48

Chromatogram

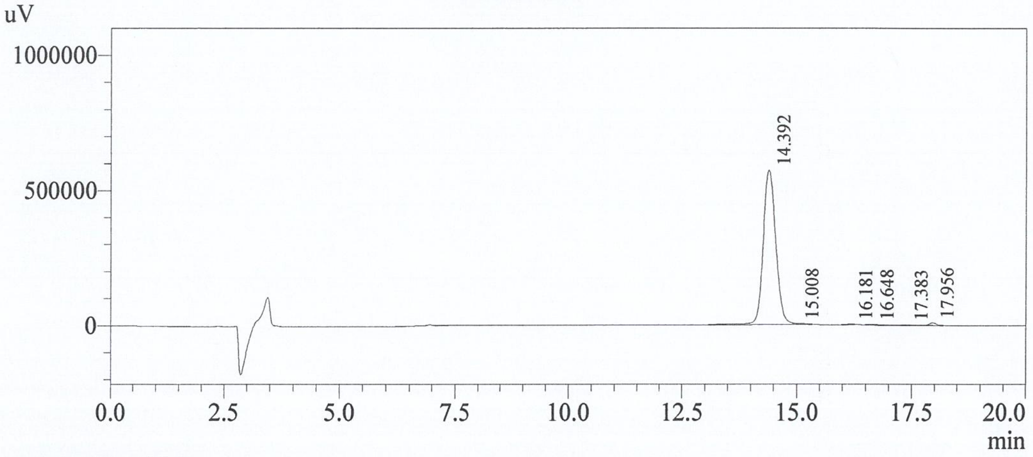

1 Det.A Ch1 / 214nm

PeakTable

Detector A Ch1 214nm

Peak#	Ret. Time	Area	Height	Area %	Height %
1	14.392	10836335	572535	98.125	97.001
2	15.008	57081	3582	0.517	0.607
3	16.181	23268	1834	0.211	0.311
4	16.648	20070	1467	0.182	0.249
5	17.383	11971	1344	0.108	0.228
6	17.956	94620	9473	0.857	1.605
Total		11043346	590236	100.000	100.000

生工生物工程（上海）股份有限公司

地址: 上海市松江区香闵路698号 Add: 698 Xiang Min Road SongJiang Shanghai China
电话/Tel: 400-821-0268 传真/Fax: 86-21-37772170
邮箱/Email: sales@sangon.com 网址/Web: www.sangon.com

MS Spectrum

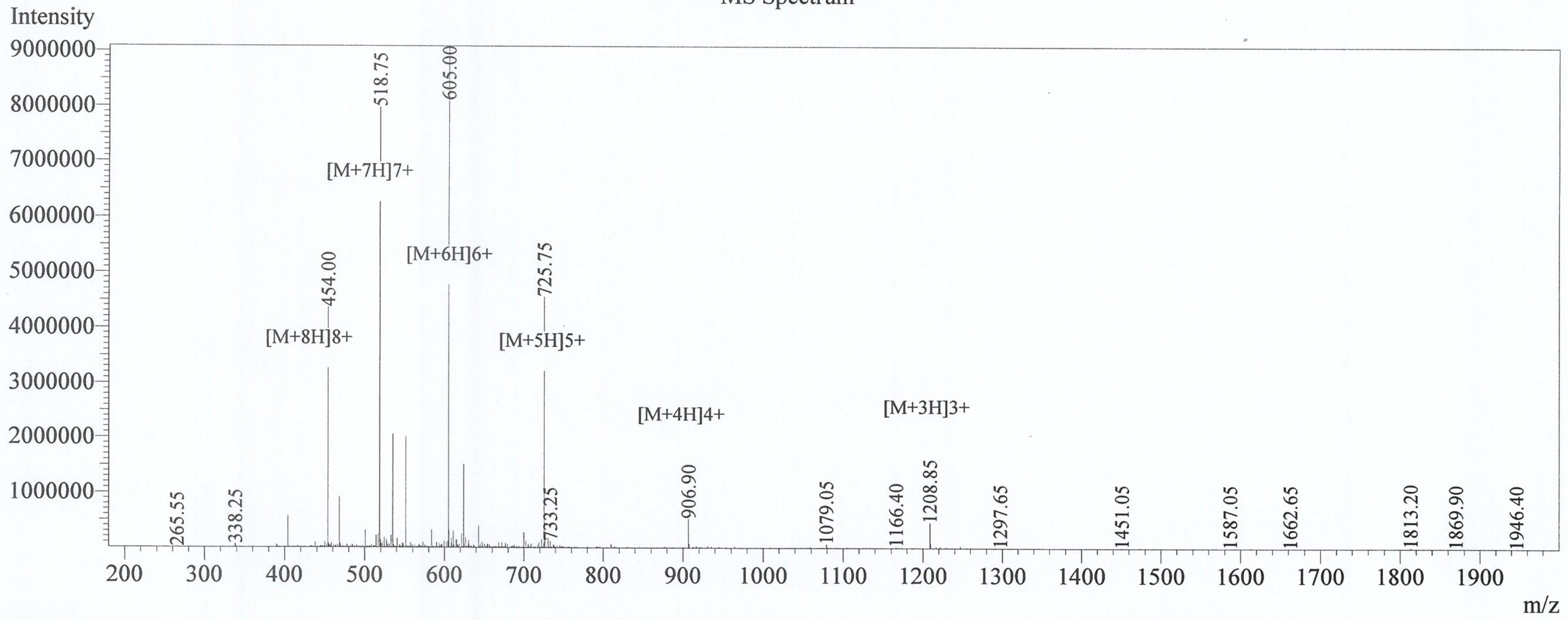

Sample Information
Dissolution method :0.1mg sample dissolved to 0.5mL by 50%ACN and 50%H2O
Date Acquired :2023/10/26 14:19:58
Injection Volume :1ul
Name :P30536-8（Fcontrol）
Sequence :FITC-Acp-YGRKKRRQRRREAKLKAFFAKSLKV
Modification :N-terminal FITC-Acp
Lot No. :P30536-8（Fcontrol）-23092501
Theoretical :3624.545
bserved :3624.00

Interface :ESI
Nebulizing Gas Flow :1.50L/min
CDL Temp :250°C
CDL Volt :0v
Block Temp :200

Prerod Bias :+4.5kv
Detector :-0.2kv
T.Flow :0.2ml/min
B.conc :50%H2O/50%MeOH

生工生物工程（上海）股份有限公司

地址：上海市松江区香闵路698号
电话/Tel: 400-821-0268
邮箱/Email: Sales@sangon.com

Add: 698 Xiang Min Road Songjiang Shanghai China
传真/Fax: 86-21-37772170
网址/Web: www.sangon.com

CERTIFICATE OF ANALYSIS

**Product Name**	P30536-8（Fcontrol）
**Catalog No.**	N/A
**Lot No.**	P30536-8（Fcontrol）-23092501
**Sequence**	FITC-Acp-YGRKKRRQRRREAKLKAFFAKSLKV
**Length**	25AA
**Modification**	N-terminal FITC-Acp
**Molecular Weight (MW)**	3624.55
**Storage**	-20°C

**Test Items**	**Specifications**	**Results**
**MW by MS**	3624.0	Conforms
**Purity by HPLC**	＞95%脱盐	98.125%脱盐
**Peptide Content**	N/A	N/A
**Acetic acid content**	N/A	N/A
**Appearance**	Yellow lyophilized powder	Conforms
**Quantity**	5mg*2vials	10.0mg

Certified by: Melinda Date 10/26/2023

Quality Assurance Department

生工® Sangon Biotech

Sample Information

Name	：P30536-9（F1）
Sequence	：FITC-Acp-YGRKKRRQRRRFKKLAEAVKFSAKL
Modification	：N-terminal FITC-Acp
Lot.No	：P30536-9（F1）-23092501
Pump A	：0.1%trifluoroacetic in 100%water
Pump B	：0.1%trifluoroacetic in 100%acetonrtrile
Total Flow	：1.0ml/min
Wavelength	：214nm
Analytical column type	：SHIMADZU shim-pack GIST(4.6*250MM*5UM)
Dissolution method	：0.1mg sample dissolved to 0.5mL by 10%ACN and 90%H2O
Acquisition Time	：2023/10/26 14:08:53
Inj.Volume	：30ul

Time	Module	Action	Value
0.01	Pumps	B.Conc	26
20.00	Pumps	B.Conc	46

Chromatogram

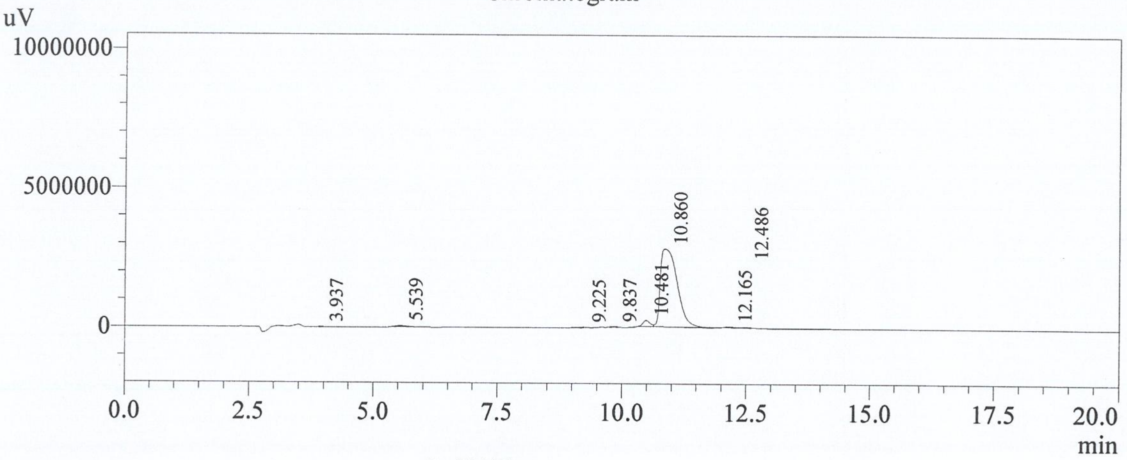

PeakTable

检测器A 214nm

Peak#	Ret. Time	Area	Height	Area %	Height %
1	3.937	28999	6554	0.042	0.209
2	5.539	479835	35342	0.695	1.130
3	9.225	87415	12173	0.127	0.389
4	9.837	180725	23525	0.262	0.752
5	10.481	2203334	218557	3.189	6.985
6	10.860	65713765	2792771	95.114	89.257
7	12.165	261653	22694	0.379	0.725
8	12.486	133494	17277	0.193	0.552
总计		69089220	3128893	100.000	100.000

生工生物工程（上海）股份有限公司

地址: 上海市松江区香闵路698号
电话/Tel: 400-821-0268
邮箱/Email: sales@sangon.com

Add: 698 Xiang Min Road SongJiang Shanghai China
传真/Fax: 86-21-37772170
网址/Web: www.sangon.com

生工® Sangon Biotech

MS Spectrum

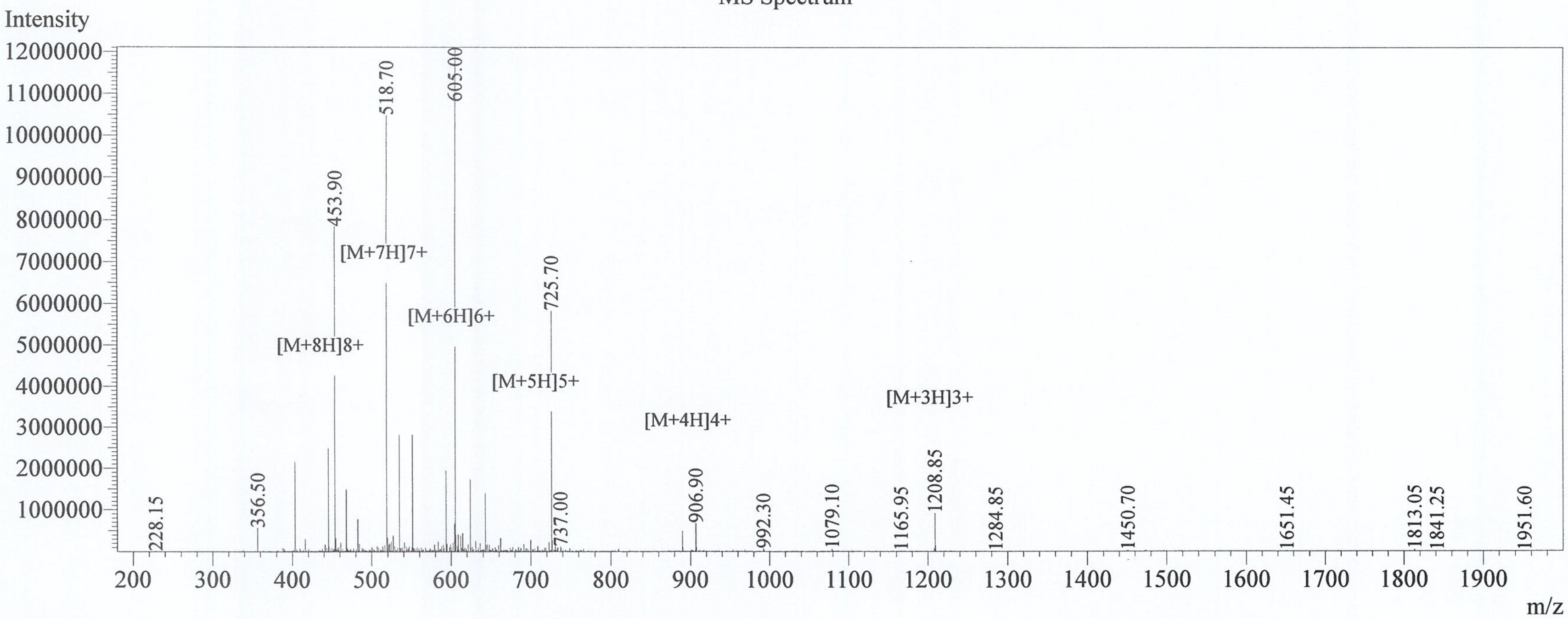

Sample Information
Dissolution method :0.1mg sample dissolved to 0.5mL by 50%ACN and 50%H2O
Date Acquired :2023/10/26 14:53:09
Injection Volume :1ul
Name :P30536-9（F1）
Sequence :FITC-Acp-YGRKKRRQRRRFKKLAEAVKFSAKL
Modification :N-terminal FITC-Acp
Lot No. :P30536-9（F1）-23092501
Theoretical :3624.545
bserved :3624.00

Interface :ESI
Nebulizing Gas Flow :1.50L/min
CDL Temp :250°C
CDL Volt :0v
Block Temp :200

Prerod Bias :+4.5kv
Detector :-0.2kv
T.Flow :0.2ml/min
B.conc :50%H2O/50%MeOH

生工生物工程（上海）股份有限公司

地址：上海市松江区香闵路698号
电话/Tel: 400-821-0268
邮箱/Email: Sales@sangon.com

Add: 698 Xiang Min Road Songjiang Shanghai China
传真/Fax: 86-21-37772170
网址/Web: www.sangon.com

生工® Sangon Biotech

多肽合成报告单

CERTIFICATE OF ANALYSIS

**Product Name**	P30536-9（F1）
**Catalog No.**	N/A
**Lot No.**	P30536-9（F1）-23092501
**Sequence**	FITC-Acp-YGRKKRRQRRRFKKLAEAVKFSAKL
**Length**	25AA
**Modification**	N-terminal FITC-Acp
**Molecular Weight (MW)**	3624.55
**Storage**	-20°C

**Test Items**	**Specifications**	**Results**
**MW by MS**	3624.0	Conforms
**Purity by HPLC**	＞95%脱盐	95.114%脱盐
**Peptide Content**	N/A	N/A
**Acetic acid content**	N/A	N/A
**Appearance**	Yellow lyophilized powder	Conforms
**Quantity**	5mg*2vials	12.0mg

Certified by: Melinda

Date 10/26/2023

Quality Assurance Department

生工® Sangon Biotech

Sample Information

Name : P30536-10（F2）
Sequence : FITC-Acp-YGRKKRRQRRRFKKFAEAVKFSAKL
Modification : N-terminal FITC-Acp
Lot.No : P30536-10（F2）-23092501

Pump A : 0.1%trifluoroacetic in 100%water
Pump B : 0.1%trifluoroacetic in 100%acetonrtrile
Total Flow : 1.0ml/min
Wavelength : 214nm
Analytical column type : SHIMADZU shim-pack GIST(4.6*250MM*5UM)
Dissolution method : 0.1mg sample dissolved to 0.5mL by 20%ACN and 80%H2O
Acquisition Time : 2023/10/26 12:28:53
Inj.Volume : 30ul

Time	Module	Action	Value
0.01	Pumps	B.Conc	27
20.00	Pumps	B.Conc	47

Chromatogram

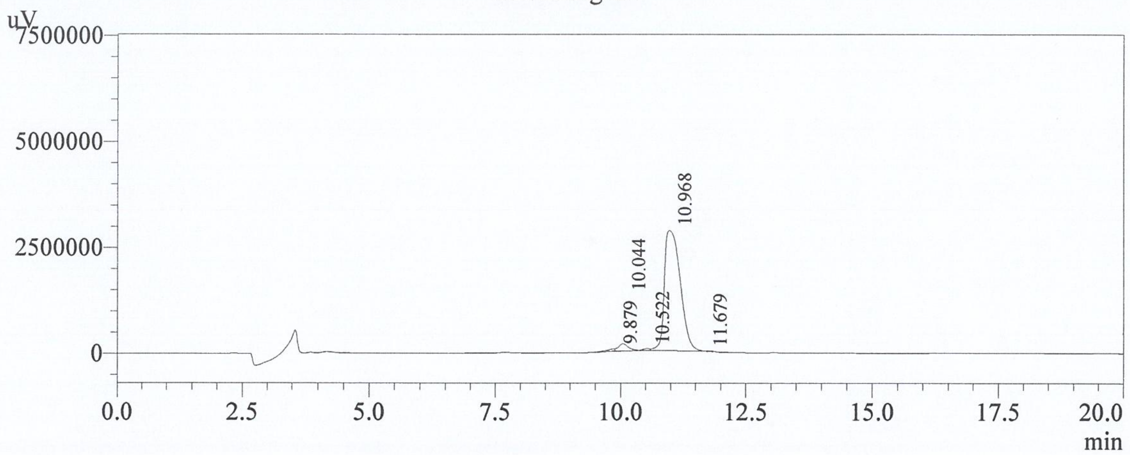

PeakTable

检测器A 214nm

Peak#	Ret. Time	Area	Height	Area %	Height %
1	9.879	574517	57123	0.859	1.837
2	10.044	2287723	171412	3.422	5.512
3	10.522	386906	41950	0.579	1.349
4	10.968	63551456	2839027	95.058	91.293
5	11.679	54931	299	0.082	0.010
总计		66855532	3109812	100.000	100.000

生工生物工程（上海）股份有限公司

地址: 上海市松江区香闵路698号
电话/Tel: 400-821-0268
邮箱/Email: sales@sangon.com

Add: 698 Xiang Min Road SongJiang Shanghai China
传真/Fax: 86-21-37772170
网址/Web: www.sangon.com

MS Spectrum

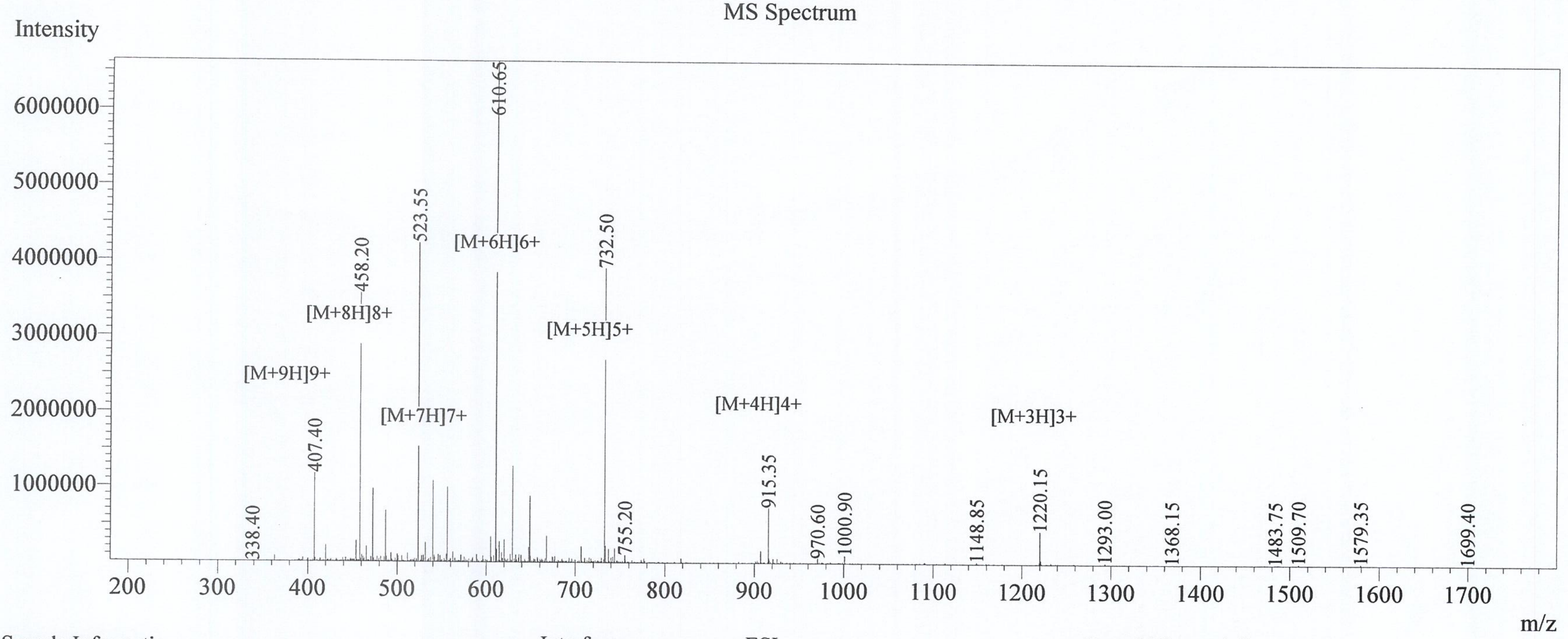

Sample Information
Dissolution method :0.1mg sample dissolved to 0.5mL by 50%ACN and 50%H2O
Date Acquired :2023/10/26 10:34:59
Injection Volume :1ul
Name :P30536-10（F2）
Sequence :FITC-Acp-YGRKKRRQRRRFKKFAEAVKFSAKL
Modification :N-terminal FITC-Acp
Lot No. :P30536-10（F2）-23092501
Theoretical :3658.562
bserved :3657.90

Interface :ESI
Nebulizing Gas Flow :1.50L/min
CDL Temp :250°C
CDL Volt :0v
Block Temp :200

Prerod Bias :+4.5kv
Detector :-0.2kv
T.Flow :0.2ml/min
B.conc :50%H2O/50%MeOH

生工生物工程（上海）股份有限公司

地址：上海市松江区香闵路698号
电话/Tel: 400-821-0268
邮箱/Email: Sales@sangon.com

Add: 698 Xiang Min Road Songjiang Shanghai China
传真/Fax: 86-21-37772170
网址/Web: www.sangon.com

CERTIFICATE OF ANALYSIS

**Product Name**	P30536-10（F2）
**Catalog No.**	N/A
**Lot No.**	P30536-10（F2）-23092501
**Sequence**	FITC-Acp-YGRKKRRQRRRFKKFAEAVKFSAKL
**Length**	25AA
**Modification**	N-terminal FITC-Acp
**Molecular Weight (MW)**	3658.56
**Storage**	-20°C

**Test Items**	**Specifications**	**Results**
**MW by MS**	3657.9	Conforms
**Purity by HPLC**	> 95%脱盐	95.058%脱盐
**Peptide Content**	N/A	N/A
**Acetic acid content**	N/A	N/A
**Appearance**	Yellow lyophilized powder	Conforms
**Quantity**	5mg*2vials	10.0mg

Certified by: Melinda Date 10/26/2023

Quality Assurance Department

生工® Sangon Biotech

Sample Information

Name	:	P30536-11（F3）
Sequence	:	FITC-Acp-YGRKKRRQRRRYKKLAEAVKYSAKL
Modification	:	N-terminal FITC-Acp
Lot.No	:	P30536-11（F3）-23092501

Pump A	:	0.1%trifluoroacetic in 100%water
Pump B	:	0.1%trifluoroacetic in 100%acetonrtrile
Total Flow	:	1.0ml/min
Wavelength	:	214nm
Analytical column type	:	SHIMADZU Inertsil ODS-SP(4.6*250MM*5UM)
Dissolution method	:	0.1mg sample dissolved to 0.5mL by 10%ACN and 90%H2O
Acquisition Time	:	2023/10/31 15:39:16
Inj.Volume	:	10ul

Time	Module	Action	Value
0.01	Pumps	B.Conc	24
20.00	Pumps	B.Conc	44

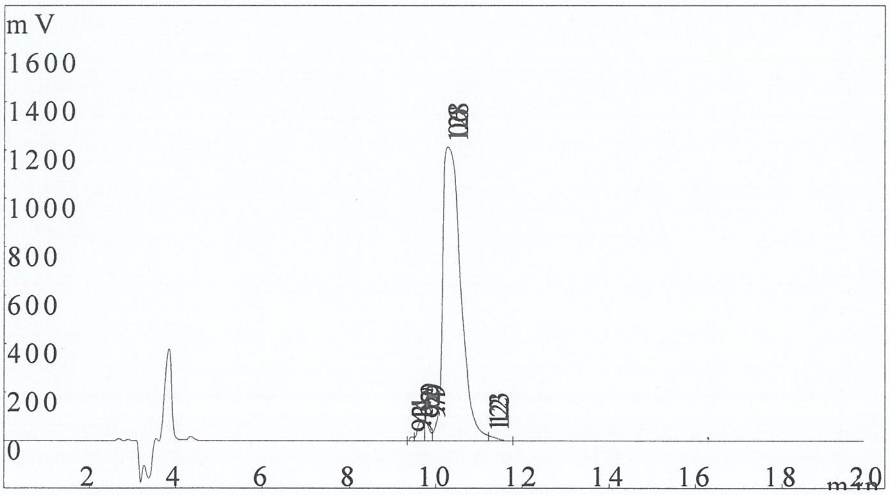

PeakTable

Detector A Ch 214nm

Peak#	Ret.Time	Area	Height	Area%	Height%
1	9.434	117948	17241	0.324	1.224
2	9.629	826539	81408	2.276	5.781
3	9.749	579905	75378	1.597	5.353
4	10.268	34534960	1212451	95.096	86.096
5	11.223	256926	21775	0.707	1.546
Total		36316278	1408253	100.000	100.000

生工生物工程（上海）股份有限公司

地址: 上海市松江区香闵路698号
电话/Tel: 400-821-0268
邮箱/Email: sales@sangon.com

Add: 698 Xiang Min Road SongJiang Shanghai China
传真/Fax: 86-21-37772170
网址/Web: www.sangon.com

生工® Sangon Biotech

MS Spectrum

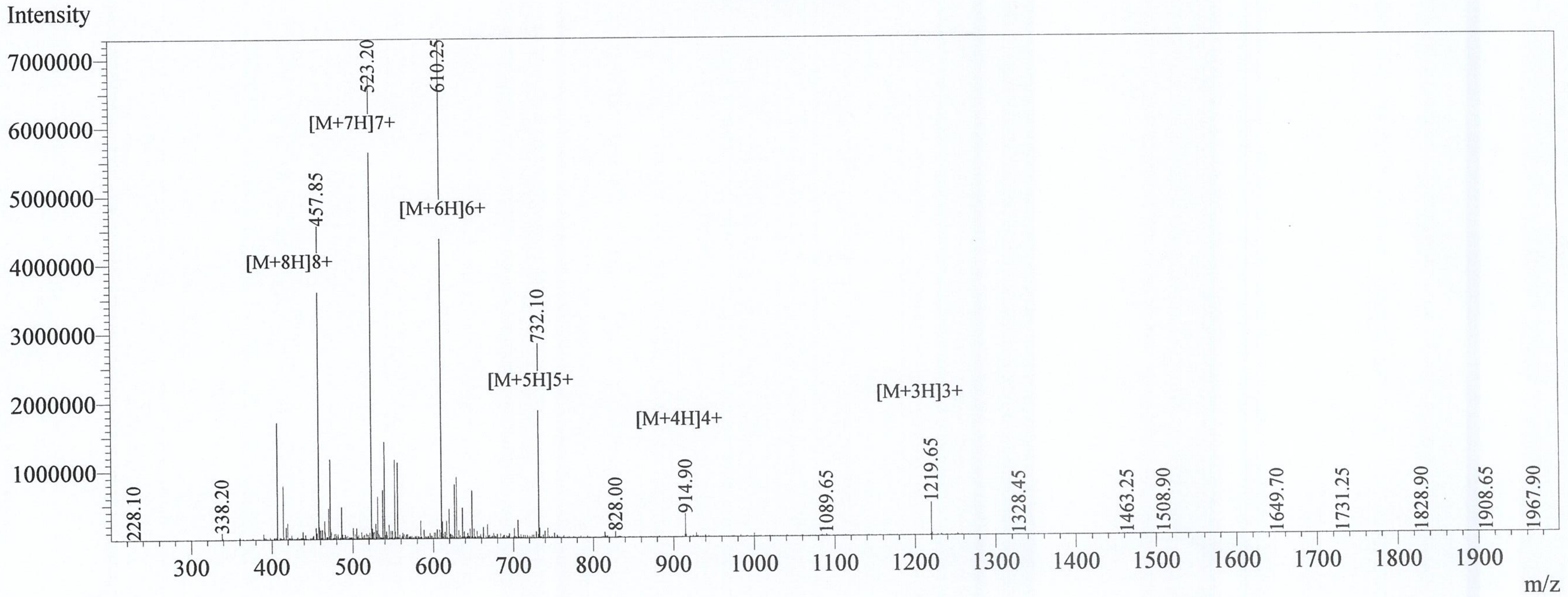

Sample Information
Dissolution method :0.1mg sample dissolved to 0.5mL by 50%ACN and 50%H2O
Date Acquired :2023/10/31 12:39:43
Injection Volume :1ul
Name :P30536-11（F3）
Sequence :FITC-Acp-YGRKKRRQRRRYKKLAEAVKYSAKL
Modification :N-terminal FITC-Acp
Lot No. :P30536-11（F3）-23092501
Theoretical :3656.543
bserved :3655.50

Interface :ESI
Nebulizing Gas Flow :1.50L/min
CDL Temp :250°C
CDL Volt :0v
Block Temp :200

Prerod Bias :+4.5kv
Detector :-0.2kv
T.Flow :0.2ml/min
B.conc :50%H2O/50%MeOH

生工生物工程（上海）股份有限公司

地址：上海市松江区香闵路698号
电话/Tel: 400-821-0268
邮箱/Email: Sales@sangon.com

Add: 698 Xiang Min Road Songjiang Shanghai China
传真/Fax: 86-2[illegible]-37772170
网址/Web: wwww.sangon.com

CERTIFICATE OF ANALYSIS

**Product Name**	P30536-11（F3）
**Catalog No.**	N/A
**Lot No.**	P30536-11（F3）-23092501
**Sequence**	FITC-Acp-YGRKKRRQRRRYKKLAEAVKYSAKL
**Length**	25AA
**Modification**	N-terminal FITC-Acp
**Molecular Weight (MW)**	3656.54
**Storage**	-20°C

**Test Items**	**Specifications**	**Results**
**MW by MS**	3655.5	Conforms
**Purity by HPLC**	＞95%脱盐	95.096%脱盐
**Peptide Content**	N/A	N/A
**Acetic acid content**	N/A	N/A
**Appearance**	Yellow lyophilized powder	Conforms
**Quantity**	5mg*2vials	10.0mg

Certified by: Melinda Date 10/31/2023

Quality Assurance Department

生工® Sangon Biotech

Sample Information

Name : P30536-12（F4）
Sequence : FITC-Acp-YGRKKRRQRRRLKKLAEALKLSAKL
Modification : N-terminal FITC-Acp
Lot.No : P30536-12（F4）-23092501

Pump A : 0.1%trifluoroacetic in 100%water
Pump B : 0.1%trifluoroacetic in 100%acetonrtrile
Total Flow : 1.0ml/min
Wavelength : 214nm
Analytical column type : SHIMADZU Inertsil ODS-SP(4.6*250MM*5UM)
Dissolution method : 0.1mg sample dissolved to 0.5mL by 10%ACN and 90%H2O
Acquisition Time : 2023/10/30 10:10:45
Inj.Volume : 10ul

Time	Module	Action	Value
0.01	Pumps	B.Conc	27
20.00	Pumps	B.Conc	47

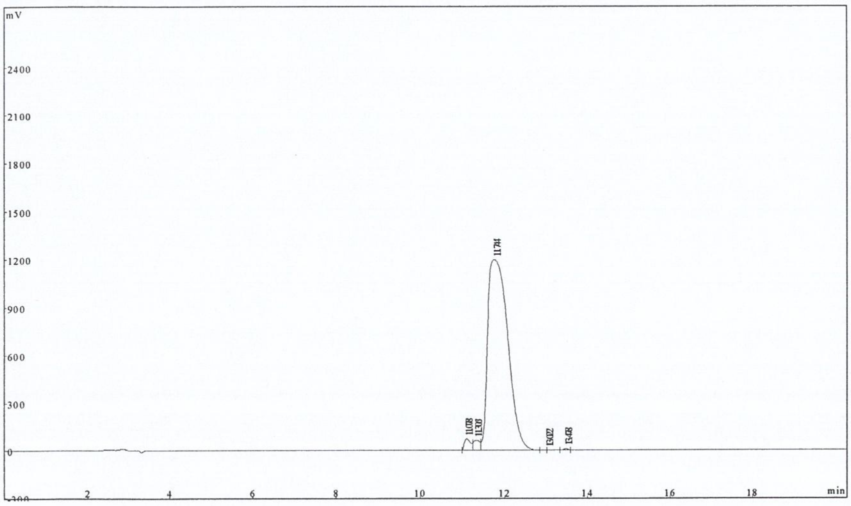

PeakTable

Detector A Ch 214nm

Peak#	Ret.Time	Area	Height	Area%	Height%
1	11.078	762422	70197	1.884	5.247
2	11.306	682223	63146	1.686	4.720
3	11.744	38948528	1196252	96.246	89.422
4	13.022	9533	0	0.023	0.000
5	13.478	65373	8164	0.161	0.611
Total		40468079	1337759	100.000	100.000

MS Spectrum

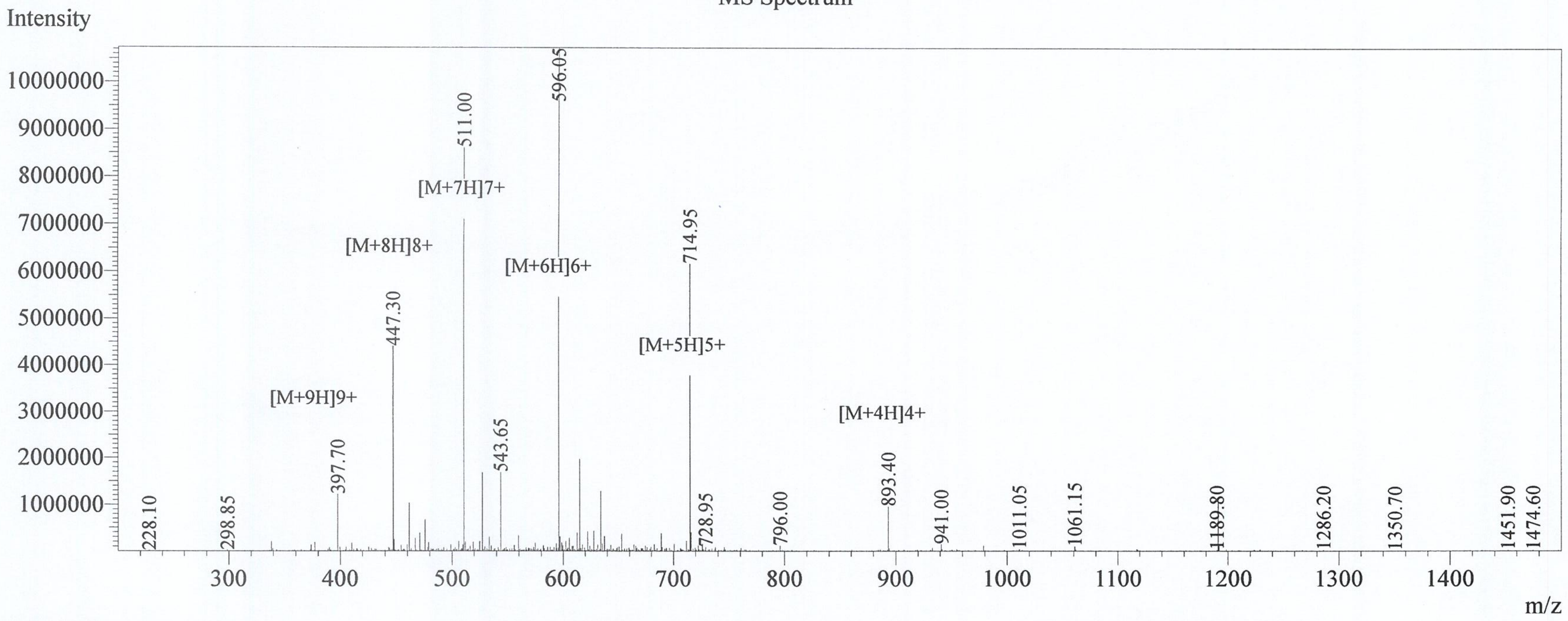

Sample Information
Dissolution method :0.1mg sample dissolved to 0.5mL by 50%ACN and 50%H2O
Date Acquired :2023/10/30 12:41:47
Injection Volume :1ul

Interface :ESI
Nebulizing Gas Flow :1.50L/min
CDL Temp :250℃
CDL Volt :0v
Block Temp :200

Prerod Bias :+4.5kv
Detector :-0.2kv
T.Flow :0.2ml/min
B.conc :50%H2O/50%MeOH

Name :P30536-12（F4）
Sequence :FITC-Acp-YGRKKRRQRRRLKKLAEALKLSAKL
Modification :N-terminal FITC-Acp
Lot No. :P30536-12（F4）-23092501
Theoretical :3570.538
bserved :3570.30

生工生物工程（上海）股份有限公司

地址：上海市松江区香闵路698号
电话/Tel: 400-821-0268
邮箱/Email: Sales@sangon.com

Add: 698 Xiang Min Road Songjiang Shanghai China
传真/Fax: 86-21-37772170
网址/Web: wwww.sangon.com

CERTIFICATE OF ANALYSIS

Product Name	P30536-12（F4）
Catalog No.	N/A
Lot No.	P30536-12（F4）-23092501
Sequence	FITC-Acp-YGRKKRRQRRRLKKLAEALKLSAKL
Length	25AA
Modification	N-terminal FITC-Acp
Molecular Weight (MW)	3570.54
Storage	-20°C

Test Items	Specifications	Results
MW by MS	3570.3	Conforms
Purity by HPLC	> 95%脱盐	96.246%脱盐
Peptide Content	N/A	N/A
Acetic acid content	N/A	N/A
Appearance	Yellow lyophilized powder	Conforms
Quantity	5mg*2vials	10.0mg

Certified by: Melinda   Date 10/30/2023

Quality Assurance Department

生工® Sangon Biotech

Sample Information

Name	:	P30536-13（F5）
Sequence	:	FITC-Acp-YGRKKRRQRRRLVKAVFFASVLM
Modification	:	N-terminal FITC-Acp
Lot.No	:	P30536-13（F5）-23092501
Pump A	:	0.1%trifluoroacetic in 100%water
Pump B	:	0.1%trifluoroacetic in 100%acetonrtrile
Total Flow	:	1.0ml/min
Wavelength	:	214nm
Analytical column type	:	SHIMADZU Inertsil ODS-SP(4.6*250MM*5UM)
Dissolution method	:	0.1mg sample dissolved to 0.5mL by 10%ACN and 90%H2O
Acquisition Time	:	2023/10/30 10:30:49
Inj.Volume	:	10ul

Time	Module	Action	Value
0.01	Pumps	B.Conc	20
20.00	Pumps	B.Conc	80

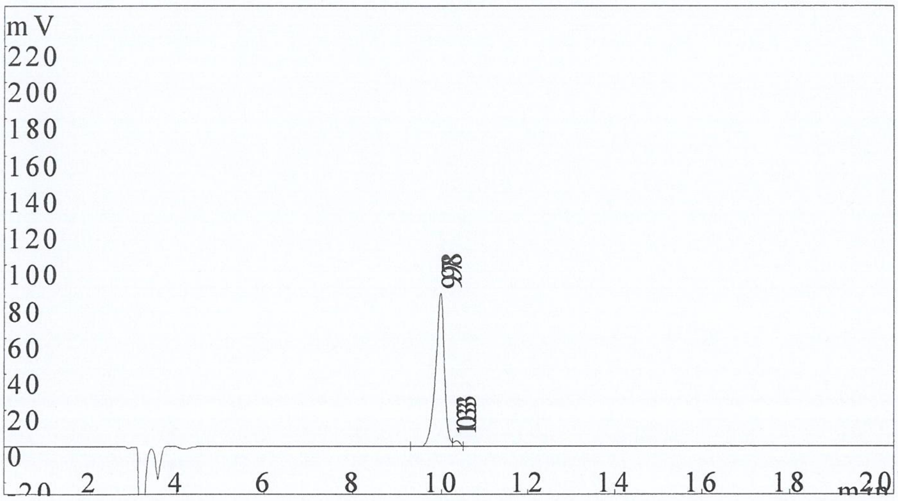

PeakTable

Detector A Ch 214nm

Peak#	Ret.Time	Area	Height	Area%	Height%
1	9.978	1118258	84123	97.426	96.429
2	10.333	29550	3115	2.574	3.571
Total		1147808	87238	100.000	100.000

生工生物工程（上海）股份有限公司

地址: 上海市松江区香闵路698号
电话/Tel: 400-821-0268
邮箱/Email: sales@sangon.com

Add: 698 Xiang Min Road SongJiang Shanghai China
传真/Fax: 86-21-37772170
网址/Web: www.sangon.com

生工® Sangon Biotech

MS Spectrum

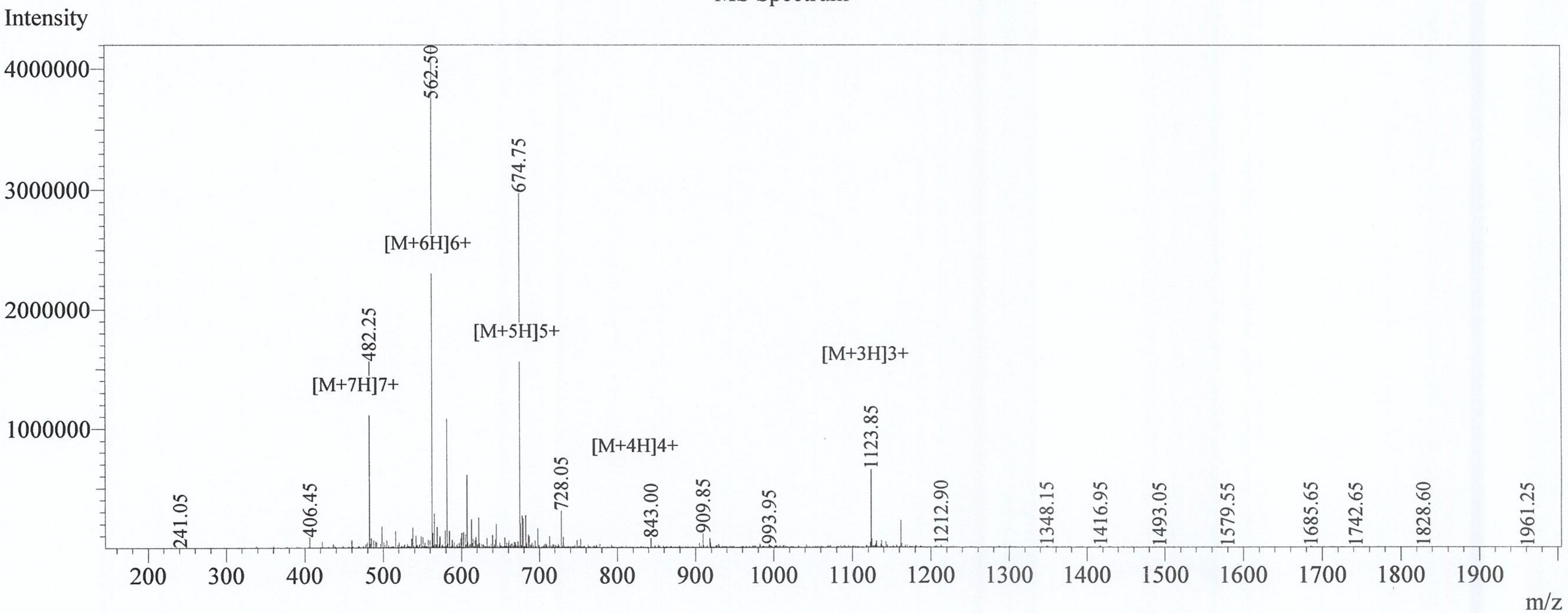

Sample Information
Dissolution method :0.1mg sample dissolved to 0.5mL by 50%ACN and 50%H2O
Date Acquired :2023/10/30 12:45:12
Injection Volume :1ul

Interface :ESI
Nebulizing Gas Flow :1.50L/min
CDL Temp :250°C
CDL Volt :0v
Block Temp :200

Prerod Bias :+4.5kv
Detector :-0.2kv
T.Flow :0.2ml/min
B.conc :50%H2O/50%MeOH

Name :P30536-13（F5）
Sequence :FITC-Acp-YGRKKRRQRRRLVKAVFFASVLM
Modification :N-terminal FITC-Acp
Lot No. :P30536-13（F5）-23092501
Theoretical :3369.305
bserved :3369.00

生工生物工程（上海）股份有限公司

地址：上海市松江区香闵路698号
Add: 698 Xiang Min Road Songjiang Shanghai China
电话/Tel: 400-821-0268
传真/Fax: 86-21-37772170
邮箱/Email: Sales@sangon.com
网址/Web: wwww.sangon.com

生工® Sangon Biotech   多肽合成报告单

CERTIFICATE OF ANALYSIS

**Product Name**	P30536-13（F5）
**Catalog No.**	N/A
**Lot No.**	P30536-13（F5）-23092501
**Sequence**	FITC-Acp-YGRKKRRQRRRLVKAVFFASVLM
**Length**	23AA
**Modification**	N-terminal FITC-Acp
**Molecular Weight (MW)**	3369.31
**Storage**	-20°C

**Test Items**	**Specifications**	**Results**
**MW by MS**	3369.0	Conforms
**Purity by HPLC**	> 95%脱盐	97.426%脱盐
**Peptide Content**	N/A	N/A
**Acetic acid content**	N/A	N/A
**Appearance**	Yellow lyophilized powder	Conforms
**Quantity**	5mg*2vials	10.0mg

Certified by: Melinda   Date 10/30/2023

Quality Assurance Department

生工® Sangon Biotech

Sample Information

Name	:	P30536-14（F6）
Sequence	:	FITC-Acp-YGRKKRRQRRRTFKEVANAVKISAS
Modification	:	N-terminal FITC-Acp
Lot.No	:	P30536-14（F6）-23092501
Pump A	:	0.1%trifluoroacetic in 100%water
Pump B	:	0.1%trifluoroacetic in 100%acetonrtrile
Total Flow	:	1.0ml/min
Wavelength	:	214nm
Analytical column type	:	SHIMADZU shim-pack GIST(4.6*250MM*5UM)
Dissolution method	:	0.1mg sample dissolved to 0.5mL by 20%ACN and 80%H2O
Acquisition Time	:	2023/10/26 11:40:17
Inj.Volume	:	10ul

Time	Module	Action	Value
0.01	Pumps	B.Conc	25
20.00	Pumps	B.Conc	45

Chromatogram

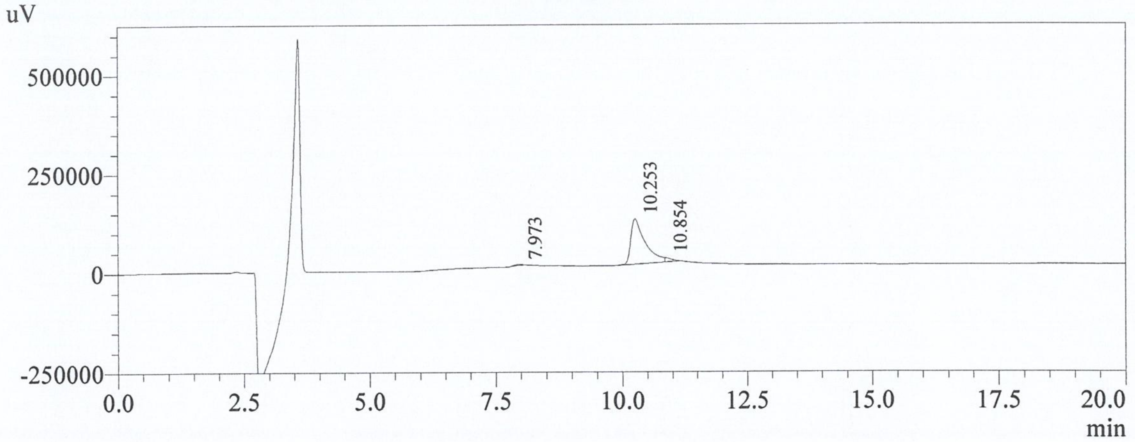

PeakTable

检测器A 214nm

Peak#	Ret. Time	Area	Height	Area %	Height %
1	7.973	22218	1993	0.942	1.574
2	10.253	2248465	114043	95.309	90.059
3	10.854	88440	10596	3.749	8.367
总计		2359124	126631	100.000	100.000

生工生物工程（上海）股份有限公司

地址: 上海市松江区香闵路698号
电话/Tel: 400-821-0268
邮箱/Email: sales@sangon.com
Add: 698 Xiang Min Road SongJiang Shanghai China
传真/Fax: 86-21-37772170
网址/Web: www.sangon.com

生工® Sangon Biotech

MS Spectrum

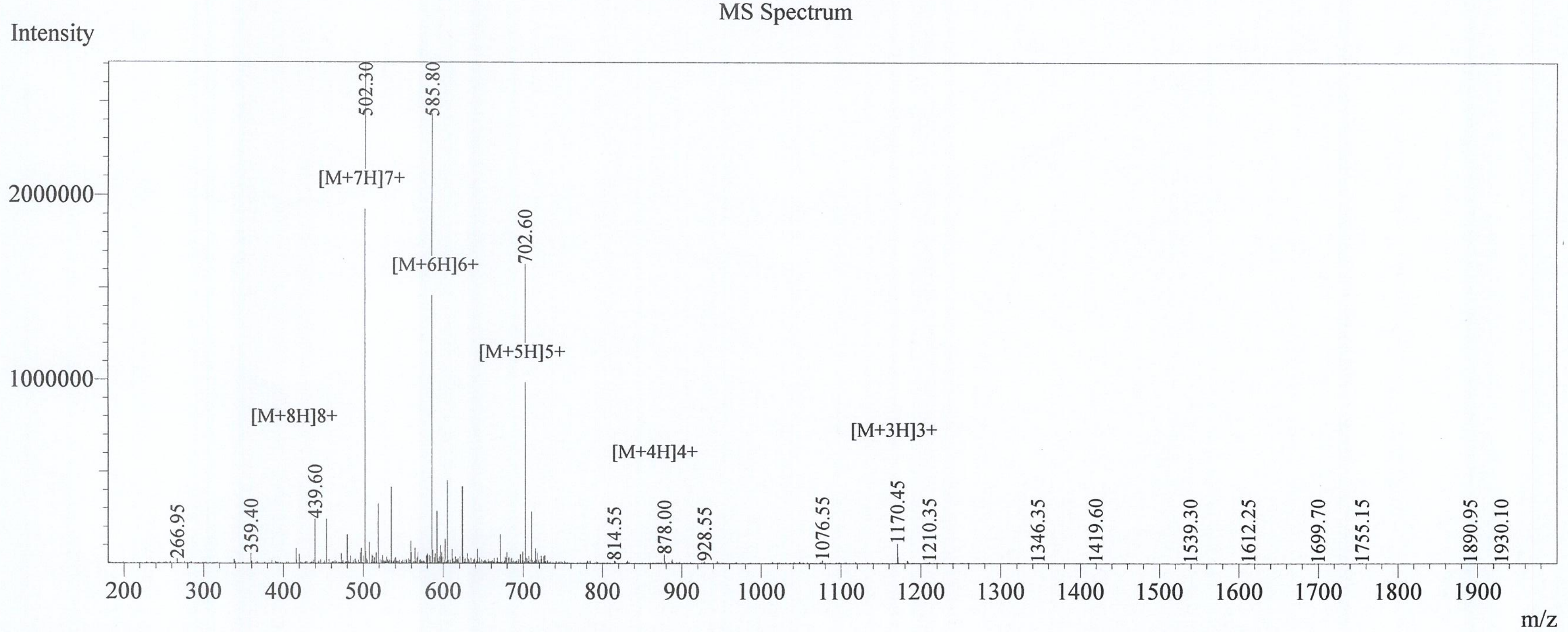

Sample Information
Dissolution method :0.1mg sample dissolved to 0.5mL by 50%ACN and 50%H2O
Date Acquired :2023/10/26 10:03:10
Injection Volume :1ul
Name :P30536-14（F6）
Sequence :FITC-Acp-YGRKKRRQRRRTFKEVANAVKISAS
Modification :N-terminal FITC-Acp
Lot No. :P30536-14（F6）-23092501
Theoretical :3509.288
bserved :3509.10

Interface :ESI
Nebulizing Gas Flow :1.50L/min
CDL Temp :250°C
CDL Volt :0v
Block Temp :200

Prerod Bias :+4.5kv
Detector :-0.2kv
T.Flow :0.2ml/min
B.conc :50%H2O/50%MeOH

生工生物工程（上海）股份有限公司

地址：上海市松江区香闵路698号
电话/Tel: 400-821-0258
邮箱/Email: Sales@sangon.com

Add: 698 Xiang Min Road Songjiang Shanghai China
传真/Fax: 86-21-37772170
网址/Web: wwww.sangon.com

生工® Sangon Biotech

多肽合成报告单

CERTIFICATE OF ANALYSIS

**Product Name**	P30536-14（F6）
**Catalog No.**	N/A
**Lot No.**	P30536-14（F6）-23092501
**Sequence**	FITC-Acp-YGRKKRRQRRRTFKEVANAVKISAS
**Length**	25AA
**Modification**	N-terminal FITC-Acp
**Molecular Weight (MW)**	3509.29
**Storage**	-20°C

**Test Items**	**Specifications**	**Results**
**MW by MS**	3509.1	Conforms
**Purity by HPLC**	> 95%脱盐	95.309%脱盐
**Peptide Content**	N/A	N/A
**Acetic acid content**	N/A	N/A
**Appearance**	Yellow lyophilized powder	Conforms
**Quantity**	5mg*2vials	10.0mg

Certified by: Melinda Date 10/26/2023

Quality Assurance Department
