# Supplementary figures and images for: K739 is preferentially targeted over K725 in the deSUMOylation process of neuronal nitric oxide synthase
Source: Front Chem. 2025 Sep 4;13:1672437. doi: 10.3389/fchem.2025.1672437 (PMC12445055; doi:10.3389/fchem.2025.1672437)

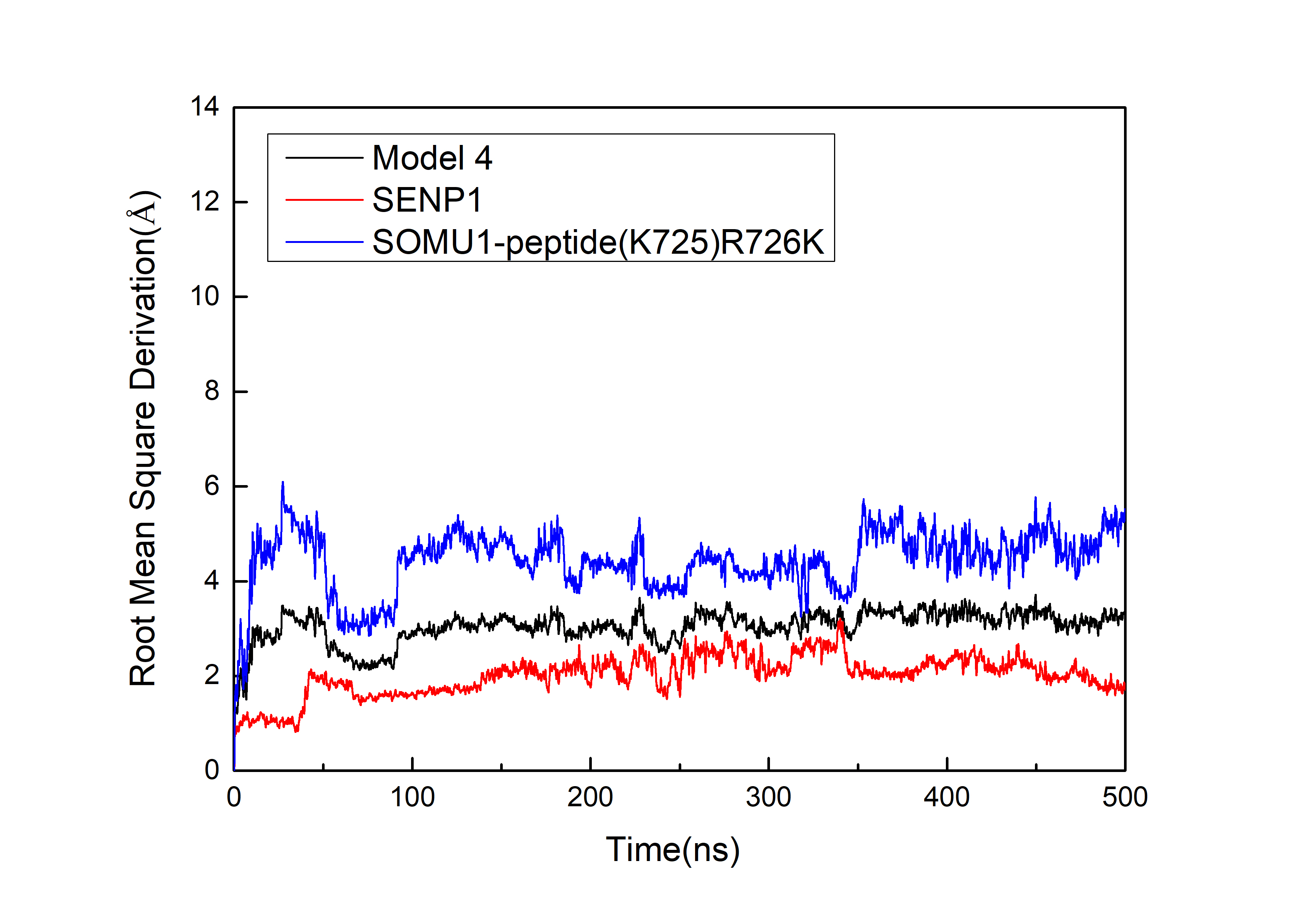

Supplement: Supplementary file 2 [file DataSheet2.zip › raw-data/725-R726K/Mutation725-RMSD.jpg]

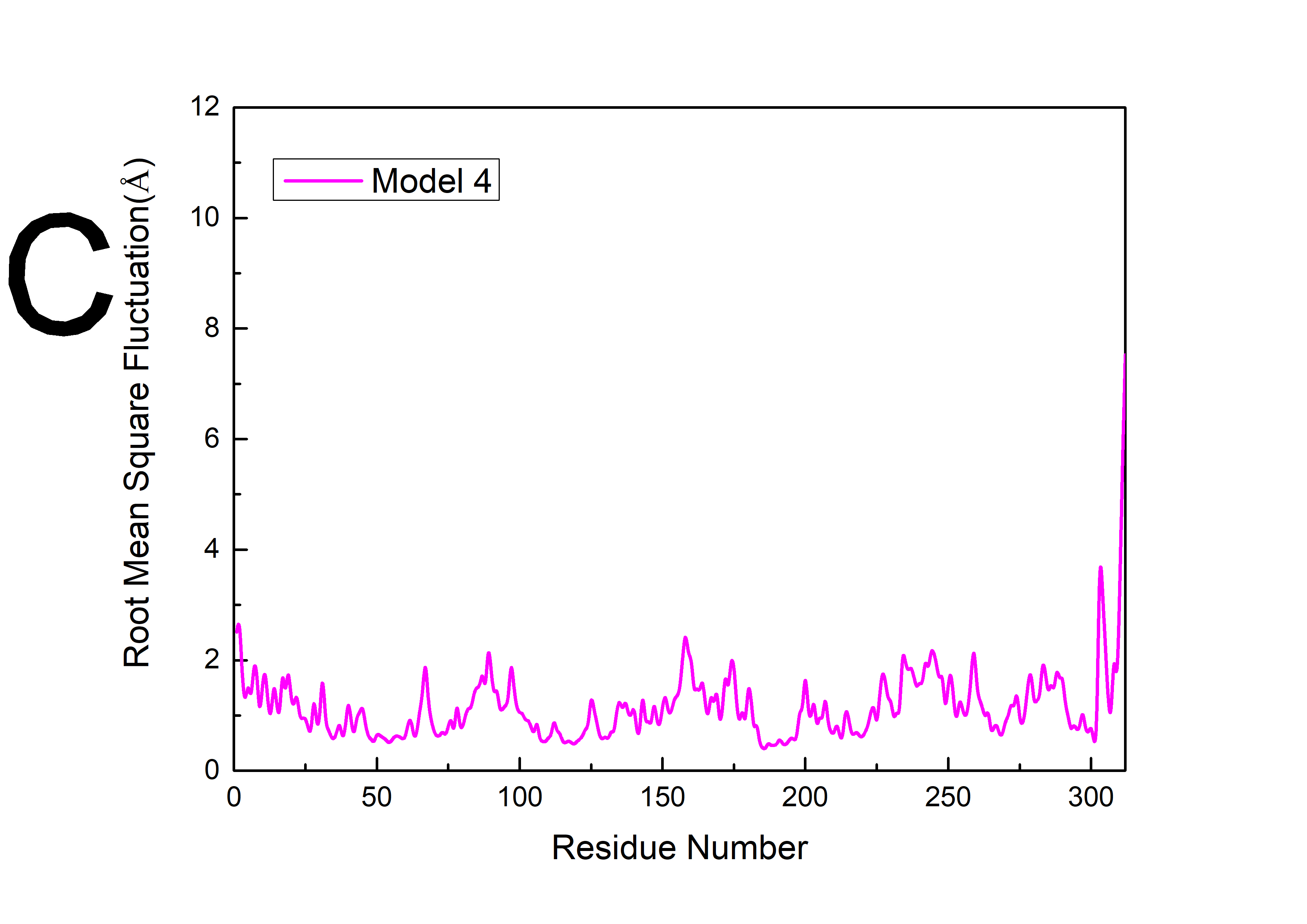

Supplement: Supplementary file 2 [file DataSheet2.zip › raw-data/725-R726K/mutation725-RMSF.jpg]

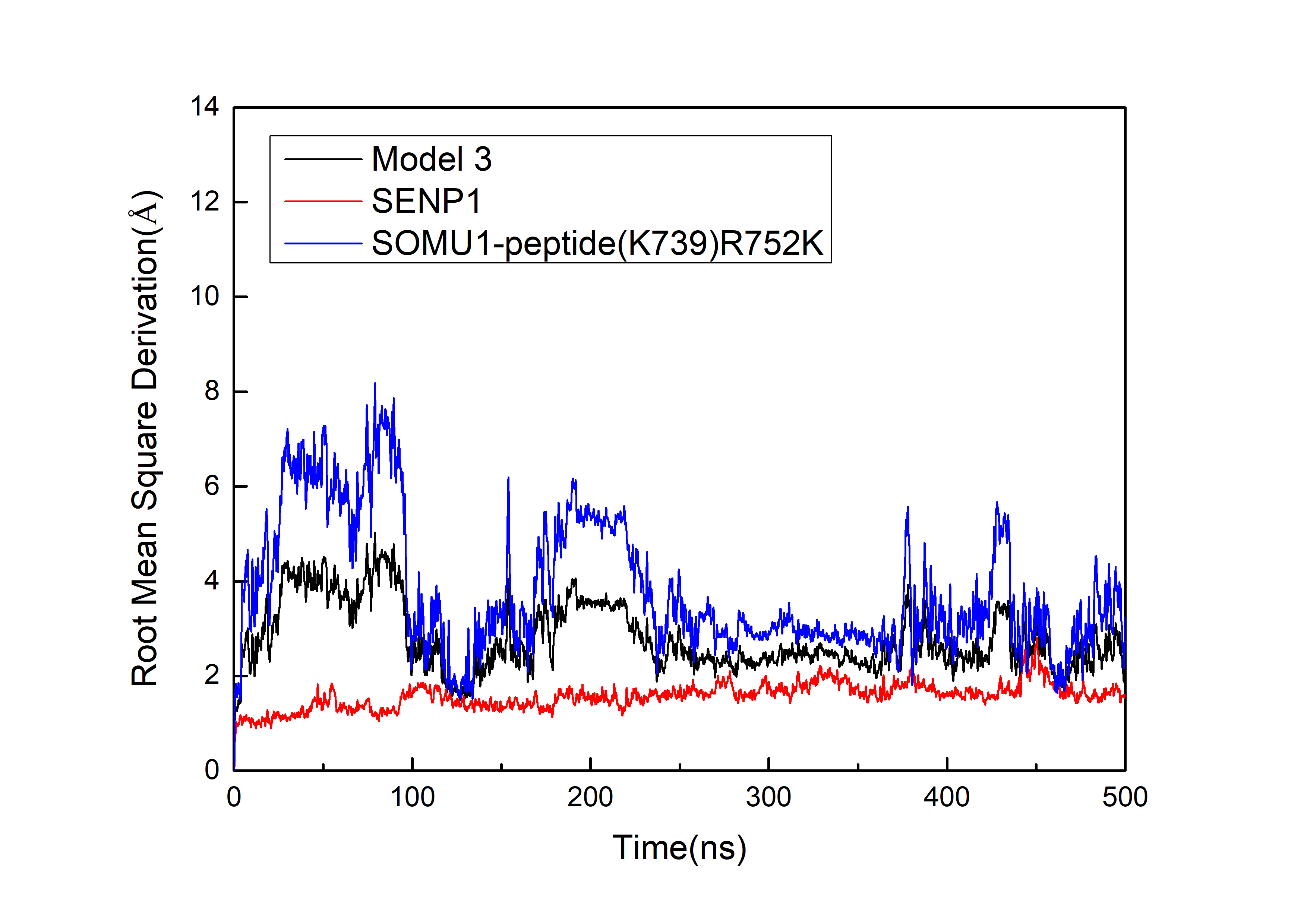

Supplement: Supplementary file 2 [file DataSheet2.zip › raw-data/739-R752K/mutation739-RMSD.jpg]
